# Examining the social networks of older adults receiving informal or formal care: a systematic review

**DOI:** 10.1186/s12877-023-04190-9

**Published:** 2023-08-31

**Authors:** Iris Szu-Szu Ho, Kris McGill, Stephen Malden, Cara Wilson, Caroline Pearce, Eileen Kaner, John Vines, Navneet Aujla, Sue Lewis, Valerio Restocchi, Alan Marshall, Bruce Guthrie

**Affiliations:** 1 Advanced Care Research Centre, Usher Institute, University of Edinburgh, Bio Cube 1, Edinburgh BioQuarter, 13 Little France Road, Edinburgh, EH16 4UX UK; 2 School of Health in Social Science, Medical School, University of Edinburgh, Doorway 6, Teviot Place, Edinburgh, EH8 9AG UK; 3 Institute for Education, Community and Society, University of Edinburgh, Old Moray House, Holyrood Road, Edinburgh, EH8 8AQ UK; 4 Edinburgh College of Art, University of Edinburgh, 74 Lauriston Pl, Edinburgh, EH3 9DF UK; 5 Population Health Science Institute, Newcastle University, Baddiley-Clark Building, Newcastle upon Tyne, NE2 4AX UK; 6 School of Informatics, University of Edinburgh (Informatics Forum, 10 Crichton St, Newington, Edinburgh, EH8 9AB UK; 7 School of Social and Political Science, University of Edinburgh, 15a George Square, Edinburgh, EH8 9LD UK; 8 47 Potterow, Bayes Centre, Edinburgh, EH8 9BT UK

**Keywords:** Older adults, Social networks, Informal care, Formal care, Healthy aging

## Abstract

**Purpose:**

To address the care needs of older adults, it is important to identify and understand the forms of care support older adults received. This systematic review aims to examine the social networks of older adults receiving informal or formal care and the factors that influenced their networks.

**Methods:**

A systematic review was conducted by searching six databases from inception to January 31, 2023. The review included primary studies focusing on older adults receiving long-term care, encompassing both informal and formal care. To assess the risk of bias in the included studies, validated appraisal tools specifically designed for different study types were utilized. Network analysis was employed to identify the grouping of study concepts, which subsequently formed the foundation for describing themes through narrative synthesis.

**Results:**

We identified 121 studies relating to the formal and informal care of older adults’ networks. A variety of social ties were examined by included studies. The most commonly examined sources of care support were family members (such as children and spouses) and friends. Several factors were consistently reported to influence the provision of informal care, including the intensity of networks, reciprocity, and geographical proximity. In terms of formal care utilization, older age and poor health status were found to be associated with increased use of healthcare services. Additionally, physical limitations and cognitive impairment were identified as factors contributing to decreased social engagement.

**Conclusion:**

This review found that older people were embedded within a diverse network. The findings of this review emphasize the importance of recognizing and incorporating the diversity of social networks in care plans and policies to enhance the effectiveness of interventions and improve the overall well-being of older adults.

**Supplementary Information:**

The online version contains supplementary material available at 10.1186/s12877-023-04190-9.

## Introduction

People around the world are living longer, with many countries experiencing rapid growth in the proportion of older adults in the population[1]. In 2021, the World Health Organization estimated that by 2030, one in six people will be aged 60 or older [1]. The shift of the population distribution towards older ages, in both high-income and low- and middle-income countries (LMICs), has raised concerns over whether and how the care and support needs of older adults are going to be met in the future [2].

To address the care and support needs of older adults, it is important to identify and understand their social networks, the forms of support within these, and the wider context in which they live and interact [3, 4]. A ‘social network’ here is defined as a social structure comprising individuals who are tied to each other through interactions and communication [5]. The structural dimensions of a social network include its content (sources of support, which can include both care from formal health and social care services and support from family and friends), size (number of social networks), intensity (strength of relationship), homogeneity (shared interests), duration (relationship duration) and frequency (interactions) [5].

Older adults are particularly susceptible to social isolation and loneliness, which can be attributed to various factors including deteriorating health, changes in the size of their social networks (such as the loss of family members or friends), and social and demographic considerations (e.g. household income, gender or ethnicity) [6–8]. On the other hand, social support, active participation, and suitable living arrangements have been suggested to play crucial roles in safeguarding the physical and mental well-being of older adults, as well as fostering active and healthy aging [9–11]. To date, existing systematic reviews on the subject of social networks in older adults have primarily focused on factors influencing their social participation in informal support and formal care settings, as well as the subsequent health implications [12–14]. However, little attention has been given to comprehensively investigate the role of informal and formal care within older adults’ social networks. A better understanding of older adults’ care networks allows efficient coordination of diverse care resources. Therefore, the aim of this study was to examine the social networks of older adults receiving informal or formal care and the factors that influenced their networks.

## Methods

This systematic review was conducted and reported based on the PRISMA 2020 statement (Supplementary Table S1)[15]. The review protocol is registered in PROSPERO (CRD42021266849).

### Search strategy

The search strategy for this review was developed in collaboration with the research team and an information specialist (Supplementary Table S2). We systematically searched electronic databases using Medline, CINAHL, Embase, PsycINFO, Web of Science and Cochrane Library from inception to January 31, 2023. Searches were run independently within each database using medical subject heading (MeSH) terms and subject headings. Relevant keywords and Boolean operators were used to capture the concepts of social networks, care, and older adults. For example, the search terms we used in CINAHL included (network* or social network* or socio-ecolog* or support network* or social interaction* or family network* or friend relationship* or friend network* or local community network* or neighbourhood network*) AND (older adult* or older person* or older people or elderly or later life or senior*) AND (Care or home care or care home or long term care or domiciliary care or carer* or paid care* or unpaid care or formal care or informal care or nursing home or community care or assisted living or retirement village).

### Eligibility criteria

We included primary studies (e.g. quantitative, qualitative, and mixed-methods studies) providing that networks of older adults were empirically examined using quantitative and/or qualitative research techniques. The population and condition of interest in this review were older adults receiving care, and studies were included if they stated their focus of interest was older adults or if the mean age of participants was 50 years or older in each study. Choosing 50 years old as the cut-off allowed us to recognise the differences in life expectancy across socio-economic groups and geographical areas, especially later life begins at 50 for people living in some deprived areas [16]. Studies that included both middle-aged and older adults but primarily focused on older adults were considered eligible. Studies with older adults receiving some form of care were included in this review. Informal care here refers to unpaid care or support provided by family members, friends, or neighbours [2]. Formal care refers to services delivered by a social and health care professional, trained carer, government, institution, or wider community [2]. As for context, we included older adults living at home receiving domiciliary care or informal care, or receiving day care, or residents in assisted living facilities or care homes, or receiving long-term care in hospitals. We excluded any study of short-term or acute care in hospitals (less than six months) and non-English articles.

### Study selection

All records were imported to Covidence for screening and deduplication. Titles and abstracts of retrieved articles were screened independently by two reviewers within the research team (KM, SM, CP, NA, CW, EK and AM). To ensure consistency in screening, two leading reviewers (KM and SM) played a supervisory role in providing guidelines and instructions to the reviewers regarding the inclusion/exclusion criteria and the screening process. Regular meetings among the reviewers were conducted to address any questions or ambiguities and to ensure a shared understanding of the screening approach. Full texts were retrieved for the remaining studies and independently screened by two reviewers (KM and SM) against the eligibility criteria. Reasons for exclusion were recorded, and any disagreement that arose during the screening process were resolved through discussion or by an arbitrator (BG) when necessary.

### Data extraction

Data extraction encompassed all study findings, with a specific focus on information relevant to the objectives of this review. The extracted data included details such as: (1) Author, (2), Publication Year, (3) Title, (4) Country, (5) Sample size, (6) Study purpose, (7) Study design, (8) Participants, (9) Setting, (10) Mean age, (11) Networks, and (11) Study findings. Major themes of each study’s findings were extracted to summarise themes/variables investigated by studies.

### Risk of bias assessment

Tools used to appraise the risk of bias of included studies were based on the study design. Qualitative studies were assessed using the Critical Appraisal Skills Programme (CASP) Qualitative Study checklist [17]. Cross-sectional studies were assessed using the critical appraisal tool developed by Downes et al[18]. Cohort studies were assessed using the CASP Cohort Study Checklist [19]. Mixed-methods studies were assessed using the Mixed-Methods Appraisal Tool (MMAT)[20]. The assessment domains included in the appraisal tools for different study designs are shown in Appendix 2. Following study appraisal against all risk of bias domains, each study was subsequently given an overall risk of bias rating: low (if the study fulfilled ≥ 70% of requirements), moderate (30–69%), or high (< 30%).

### Data analysis

Descriptive statistics were used to summarise the characteristics of individual studies. We used frequency tables to describe categorical data. Measures of mean and standard deviation were used to describe numeric data (e.g. mean age of participants).

Due to the variability of findings across included studies, it was not possible to pool the results using meta-analysis. Various concepts relevant to social networks of older adults and their associations were explored by included studies. The analysis and results are mainly qualitative, but to ensure transparency in the analytical process, network analysis was employed to identify the grouping of study concepts and support the thematic findings [21]. The Louvain optimization algorithm was utilized to detect communities, which helped identify major themes by grouping densely connected nodes or concepts [22]. This approach was chosen because it allowed for a more objective grouping of the multitude of concepts identified in this review and facilitated the understanding of their interconnections [22]. Furthermore, this method enabled the identification of closely connected concepts that formed distinct themes or communities, as well as determining the number of communities or themes within the network of concepts being studied [22] (Supplementary Box S1). The study concepts within each community served as a framework for describing the identified themes using narrative synthesis. While this particular method may not have been previously employed in a review, it offers valuable insights and advantages in terms of understanding the interconnectedness and organization of concepts within a given topic.

The concepts that exerted influence on the social networks of older adults, as identified through both quantitative and qualitative studies, were systematically coded from the original texts. To account for the heterogeneity across the included studies, network analysis and network diagrams were employed to document and visualize the links or relationships (edges) between these concepts (nodes). In the network diagrams, the thickness of the links represented the number of citations related to the relationships between the concepts. Given that the majority of the included studies were cross-sectional and did not allow for causal inferences, undirected networks (without inferring causal directions) were used to summarize and visually present the findings. This approach ensured a comprehensive and visual representation of the relationships between the identified concepts within the social networks of older adults.

The analysis was stratified based on whether the care provided was formal or informal. Concepts associated with formal care services, such as healthcare use, were classified under the formal care group, while other links were categorized under the informal care group. This stratification allowed for the identification of differences in influential concepts and links between formal and informal care provision. To assess the robustness of the findings, a sensitivity analysis was conducted, removing studies with a high risk of bias, to determine if the conclusions remained consistent. By implementing this stratified analysis and sensitivity analysis, the study aimed to provide a comprehensive understanding of the distinct factors and relationships within formal and informal care contexts. Analyses were conducted using RStudio 4.2.1.

## Results

**Fig. 1:**
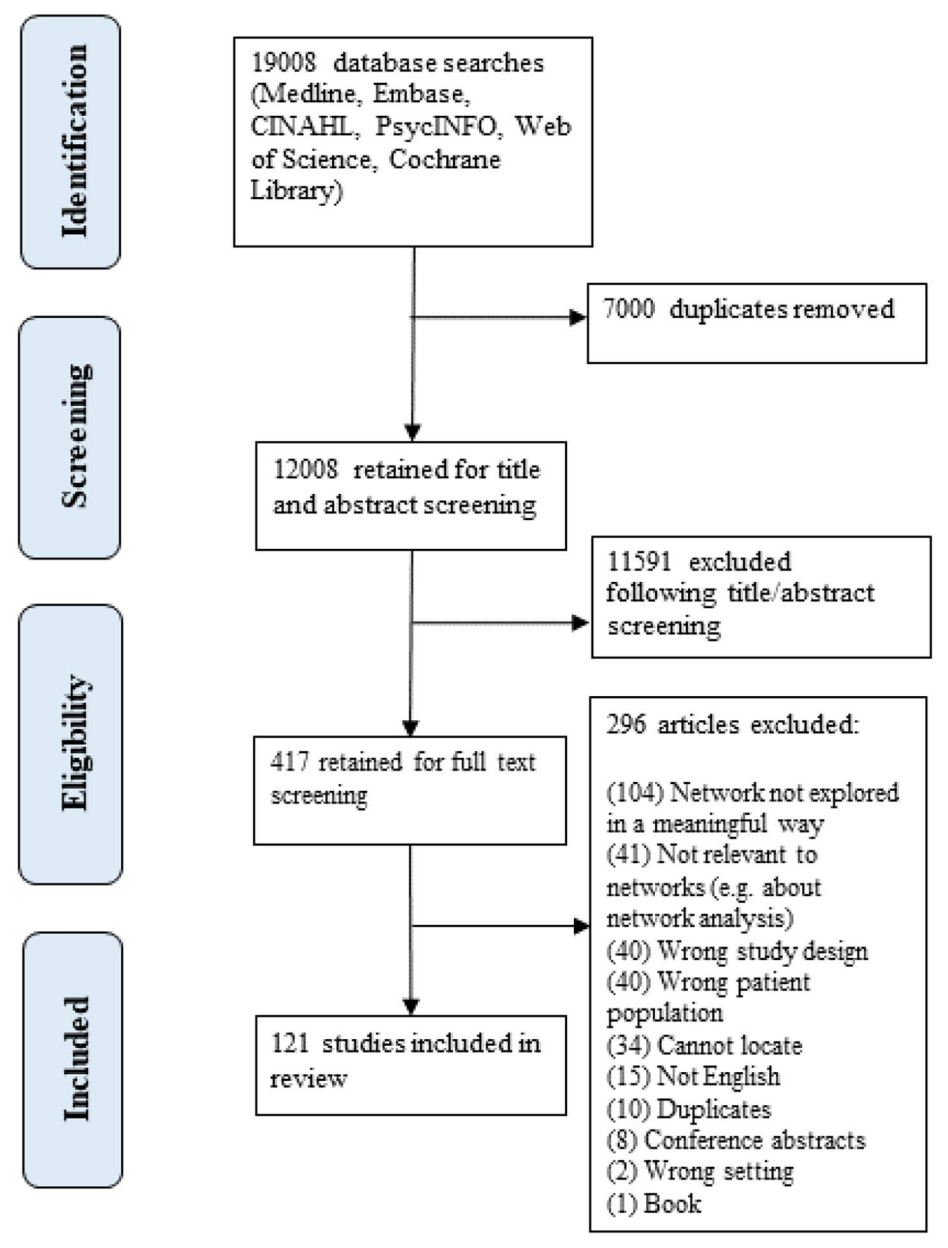
PRISMA flow diagram

19,008 records were identified from the systematic searches, of which 7000 duplicates were removed and 12,008 were retained for title, abstract and full-text screening against eligibility criteria. 11,887 were further excluded, leaving 121 included for the final review. The screening process and rationales for exclusion are reported in Fig. 1. A number of studies were excluded from this review for various reasons. Some of the excluded papers focused on carers’ social networks, interventions of different types, or were conducted in narrow populations of older adults, such as those with dementia. Additionally, some studies did not extensively or meaningfully explore the networks of older adults, while others were relevant to acute settings. Although these studies hold considerable value, they fell outside the scope of this particular review. Here are a few examples [23–27] of the excluded studies, which help illustrate the types of studies that did not meet the inclusion criteria.

Of the 121 studies (Table 1 and Supplementary Table S3), 83 were quantitative (68.6%), 34 were qualitative (28.1%), and four were mixed-methods (3.3%). Studies were published between 1981 and 2022, with over half of included studies (62.8%) published between 2011 and 2021. Eighty-four (89.3%) studies were from high-income countries, and 13 (10.7%) from low- and middle-income countries. Forty-three (35.5%) were from North America, 40 (33.1%) from Europe, 26 (21.5%) from Asia, seven (5.8%) from Australasia and four (3.3%) from South America. The majority of included studies (110 studies, 90.9%) examined networks of the older adult general populations, with small numbers focusing on older adults who were lesbian, gay, bisexual, and transgender (LGBT) (3 studies, 2.5%), who had low socio-economic status (2 studies, 1.6%) or certain condition or disability (6 studies, 5.0%). The mean age of participants in each study ranged from 57 to 85 (mean across all studies 73.7 years, SD: 6.5).

**Table 1:** Study characteristics

Variable	Number of studies (%)
Publication year	
2011–2021 2001–2010 1991–2000 1981–1990	76 (62.8%) 27 (22.3%) 11 (9.1%) 7 (5.8%)
Continent	
North America Europe Asia Australasia South America Multiple continents	43 (35.5%) 40 (33.1%) 26 (21.5%) 7 (5.8%) 4 (3.3%) 1 (0.8%)
Income country	
High income	108 (89.3%)
Low or Middle income	13 (10.7%)
Study design	
Quantitative Qualitative Mixed methods	83 (68.6%) 34 (28.1%) 4 (3.3%)
Study population	
Older adults	110 (90.9%)
Older adults with physical, mental or learning disability LGBTQ older adults Older adults with low socio-economic status	6 (5.0%) 3 (2.4%) 2 (1.7%)
Mean age of participants in each study	
Range Mean (SD)	57–85 73.7 (6.5)
Study settings	
Community Care institutions	105 (86.8%) 16 (13.2%)

### Social networks examined by studies

A wide range of social ties, linking older adults with other individuals and the broader society, were examined across the included studies (Table 2). Around three fourths (72.7%) of the studies examined the support provided to older adults by their family members. Children were most frequently examined as a source of family support (38.0% of studies), followed by spouse (33.9%), other close relatives (22.3%) (e.g. children-in-law, nieces and nephews), and siblings (5.0%). Other sources of support examined included friends (60.3%), neighbours (25.6%), church members (9.9%), co-residents (7.4%), pets (2.5%), health and social care providers (24.8%), and community (e.g. support groups and third sector) (38.8%). Among the social networks investigated, close family members, such as spouses and children, emerged as the most preferred and important source of support for older adults receiving care [28–31]. In contrast, three studies showed that friends were the primary source of support for LGBT older adults, which can be attributed to the frequently distant relationships they have with their family members [32–34]. In terms of types of support, family and friends predominantly offered emotional and financial support to older adults, while support from health and social care professionals tended to be more instrumental and functional in nature [35–40].

**Table 2:** Types of networks examined by studies

Category	Sub-category^a^	Social networks	No of studies examining social networks (%)
Older adults	All (n = 121)	Family^b^ Children Spouse Other relatives^c^ Siblings	88 (72.7%) 46 (38.0%) 41 (33.9%) 27 (22.3%) 6 (5.0%)
		Friends	73 (60.3%)
		Neighbours	31 (25.6%)
		Church members	12 (9.9%)
		Co-residents	9 (7.4%)
		Work colleague	7 (5.8%)
		Pets	3 (2.5%)
		Health and social care providers	30 (24.8%)
		Community (e.g. support groups, voluntary sector)	47 (38.8%)
Setting	Older adults living in community (n = 105)	Family^b^ Children Spouse Siblings Other relatives^c^	80 (76.2%) 46 (43.8%) 40 (38.1%) 5 (4.8%) 25 (23.8%)
		Friends	64 (61.0%)
		Neighbours	30 (28.6%)
		Church members	12 (11.4%)
		Work colleagues	7 (6.7%)
		Co-residents (in a retirement complex)	2 (1.9%)
		Health and social care providers	25 (23.8%)
		Community	41 (39.0%)
	Older adults living in care institution (n = 16)	Family^b^ Spouse Siblings Other relatives^c^	8 (50%) 1 (6.3%) 1 (6.3%) 2 (12.5%)
		Friends	9 (56.3%)
		Co-residents	7 (43.8%)
		Neighbours	1 (6.3%)
		Pets	3 (18.8%)
		Health and social care providers	5 (31.3%)
Country income	High-income countries (n = 108)	Family^b^ Children Spouse Siblings Other relatives^c^	76 (70.4%) 41 (38.0%) 37 (34.3%) 6 (5.6%) 26 (24.1%)
		Friends	64 (59.3%)
		Neighbours	27 (25.0%)
		Church members	9 (8.3%)
		Co-residents	9 (8.3%)
		Pets	3 (2.8%)
		Health and social care providers	27 (25.0%)
		Community	43 (39.8%)
	Low- or Middle-income countries (n = 13)	Family^b^ Children Spouse Other relatives^c^	12 (92.3%) 5 (38.5%) 4 (30.8%) 1 (7.7%)
		Friends	9 (69.2%)
		Neighbours	4 (30.8%)
		Church members	3 (23.1%)
		Health and social care providers	3 (23.1%)
		Community	4 (30.8%)

a. Supplementary Table S8 shows additional subgroups

b. Some studies examine ‘family support’ without specifying different types of family members

c. Other relatives includes daughters and sons in law, nephews and nieces

Compared with older adults living in community, co-residents were more frequently examined as a source of support for older adults living in a care institution (7/16 studies, 43.8%). The support received by older adults, as examined in studies published between 2001 and 2022, exhibited greater diversity compared to studies published between 1981 and 2000. In particular, support from health and social care professionals and community engagement received less attention in studies conducted prior to 2001 (6/18 studies, 33.3%). The majority of studies conducted in LMICs (12/13 studies, 92.3%) primarily focused on family care support, with limited exploration of other sources of support. Few studies from LMICs examined older adults’ engagement with community services (4/13, 30.8%) compared with those from high-income countries (43/108, 39.8%).

### Concepts relevant to older adults’ networks and links between concepts reported by studies

Several concepts and links between concepts relevant to older adults’ networks were identified by included studies (Table 3). The definitions of the concepts are documented in Supplementary Table S4 and S5. Of the links identified, thirteen studies reported that reciprocity (meaning mutual exchange of support or sharing) was positively related to the strength of relationships between older adults and their social networks [29, 41–52]. Twelve studies found that living with or close by their social networks was linked to increased access to informal care support by older adults [31, 49, 53–62]. Eleven studies reported that active social engagement among older adults was associated with increased network diversity [28, 46, 60, 63–70]. The creation of social space/opportunities was found by nine studies to be associated with increased social/community engagement of older adults (which was more commonly examined in Western countries, 7/9 studies) [45, 71–78]. A social space is defined as a recreational space where people can gather and interact. Active engagement in community activities was associated with older adults’ mental wellbeing and connectedness with the society [48, 64, 74, 77–82]. Other less frequently-reported links are summarised narratively in the themes section and in Supplementary Table S6.

**Table 3:** Most common links between concepts identified by included studies

Links between		Number of studies reporting the link	Findings	Study type
Concept01	Concept02			
Reciprocity/Mutuality	Intensity of networks	13	Reciprocity/mutuality was positively linked to the strength of relationship between older adults and their care networks	Qualitative studies: 9 [41–49] Quantitative studies: 2 [50, 51] Mixed methods: 2 [29, 52]
Geographical/Physical proximity	Informal care support	12	The shorter the distance between older adults and their networks, the higher likelihood of receiving informal care from them	Qualitative studies: 5 [49, 53–56] Quantitative design: 5 [31, 57–62]
Social engagement	Network diversity	11	Active social engagement was linked to increased network diversity	Qualitative studies: 4 [28, 46, 63, 64] Quantitative studies: 6 [60, 65–70]
Social space	Social engagement	9	Creation of social space and social opportunity was positively linked to older adults’ social engagement	Qualitative studies: 8 [45, 71–77] Mixed methods: 1 [78]
Social engagement	Mental wellbeing	9	Engaging in community activities was positively linked to older adults’ mental wellbeing and a sense of connectedness with the society	Qualitative studies: 5 [48, 64, 74, 77, 79] Quantitative studies: 2 [80–82] Mixed methods: 1 [78]
Frequency of contact	Intensity of networks	8	An increase in frequency of contact was linked to deepening relationships and emotional closeness. On the other hand, limited physical and telephone contacts were reported as a barrier to building rapport and receiving support	Qualitative studies: 4 [40, 53, 57, 92] Quantitative studies: 3 [50, 93, 94] Mixed methods: 1 [29]
Age	Formal care support	8	Older age was associated with the increased use of or need for formal care support	Qualitative studies: 1 [57] Quantitative studies: 6 [84–86, 89, 94, 106, 109]
Geographical/Physical proximity	Intensity of networks	8	The geographical distance between older adults and their care networks was negatively associated with older adults’ social ties.	Qualitative studies: 4[53, 56, 71, 74] Quantitative studies: 4 [61, 142, 146, 152]
Health status	Formal care support	7	Poor health status increased the likelihood of receiving formal care services	Qualitative studies: 2 [57, 76] Quantitative studies: 5 [58, 86–88, 106]
Formal care support	Intensity of networks	7	Engaging in formal care services (health and social care services) was positively linked to social connectedness	Qualitative studies: 6 [41, 48, 49, 64, 72, 73] Quantitative studies: 1 [87]
ADL limitations/ Physical disability	Social/Community engagement	7	ADL limitations were negatively associated with social engagement	Qualitative studies[56, 64, 71, 74, 76] Quantitative studies[80, 82]
Size of networks	Mental wellbeing	7	Having more support was positively associated with mental wellbeing	Quantitative studies[7, 39, 93, 95–98]

**Fig. 2:**
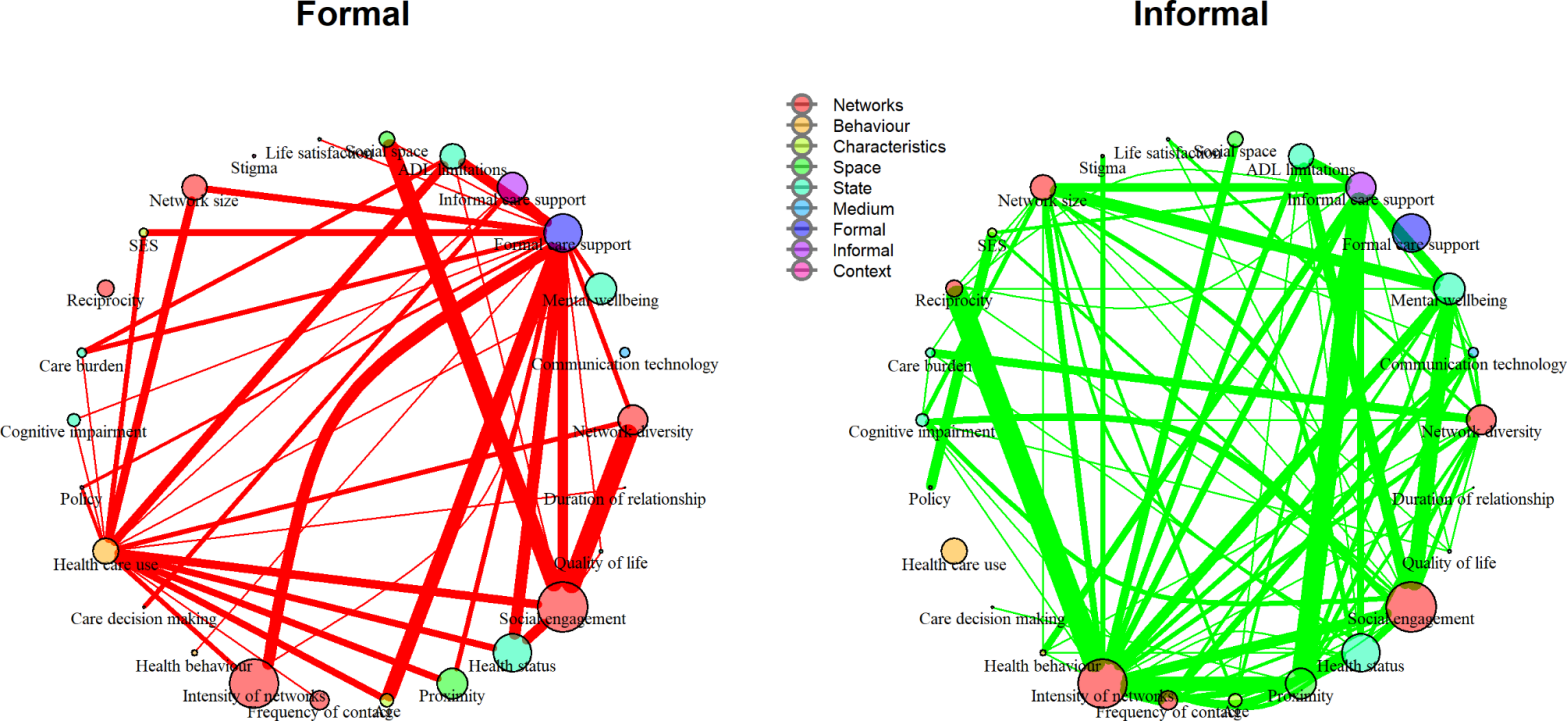
Links between concepts influencing the networks of older adults receiving formal and/or informal care. Footnote: The size of the nodes is proportional to eigenvector centrality value (Supplementary Table S5). The line thickness is proportional to citation frequency (Supplementary Table S6).

When stratified by formal and informal care groups (Fig. 2), healthcare use was linked with the greatest number of concepts in the formal care group where increases in healthcare use were associated with more physical limitations [57, 83–86], poor health status [76, 86–88], old age [84–86, 89], small network size [69, 86, 88, 90, 91] and lack of social engagement [86]. In the formal care group, the links most frequently reported were those between social engagement (defined as older adults’ involvement with community services in the formal care group) and network diversity [28, 46, 60, 63–70], and between social space and community engagement [45, 71–78]. On the other hand, in the informal care group, intensity of networks was linked with the greatest number of concepts (including reciprocity, geographical proximity and social engagement). The strongest single links found were those between reciprocity and network intensity [29, 41–52], between geographical proximity and informal care support [31, 49, 53–62], and between social engagement (defined as social interactions in the informal care group) and mental wellbeing [48, 64, 74, 77–82].

### Concepts within the three theme groups identified

**Fig. 3:**
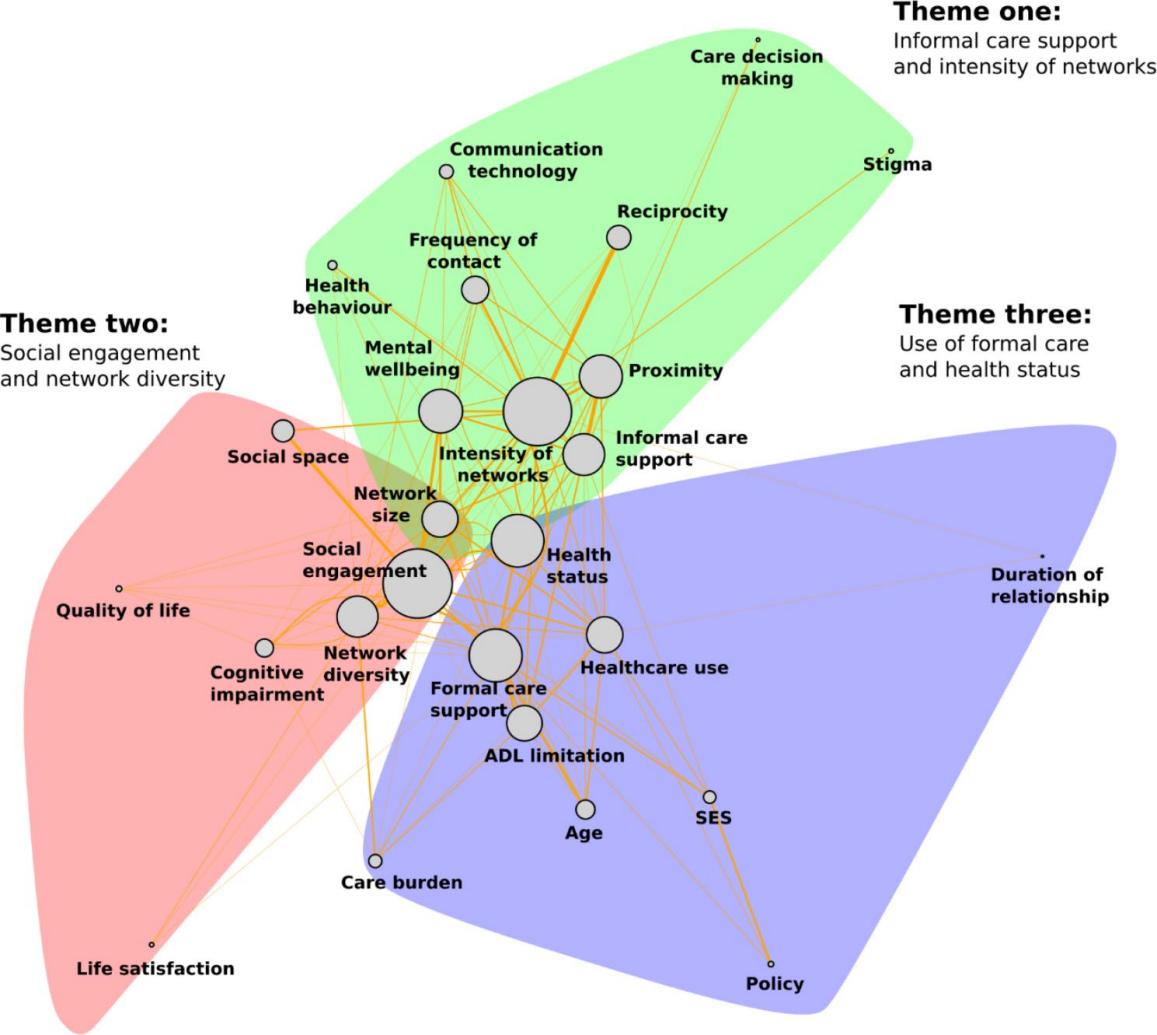
Three major themes identified to influence older adults’ social networks Footnote: Theme 1 revolves around informal care support and the intensity of networks, which were influenced by factors such as geographical proximity, frequency of contact, reciprocity (mutual interests), the use of communication technology, stigma, and mental wellbeing. Moreover, informal support significantly impacted older adults’ health behaviour and care decision-making. Theme 2 focuses on social engagement, wherein social participation was closely linked to older adults’ network diversity, quality of life and life satisfaction. The creation of social spaces facilitated such engagement, while limitations in activities of daily living (ADL) and cognitive impairment hindered social involvement. Theme 3 centers around the utilization of formal care support. Factors such as older adults’ health status, advanced age, socio-economic status, care burden and policy influenced their use of formal services. Notably, the modularity score, approximately 0.19, indicates that the groups were not distinctly separate from one another but interconnected.

Three theme groups were identified using the Louvain method (Fig. 3 and Supplementary Table S7, and references are in Supplementary Table S6). The first theme revolved around informal care support and the intensity of networks. Our findings indicated that older adults who resided in close proximity to their informal care networks experienced a higher level of informal care support [3, 31, 49, 53–62]. Mutual interests/sharing (reciprocity) [29, 41–52, 71] and maintaining daily contact [29, 40, 50, 53, 57, 92–94] were positively associated with the formation of strong bonds within the network between older adults and their support system. For those living far away from their family/friends, communication technology (e.g. telephone, email and mobile apps) provided a means for older adults to connect with their social networks and sustain meaningful relationships [56, 63, 92]. A significant relationship was found between the extent of support received by older adults and improved mental wellbeing [7, 39, 93, 95–98]. Informal caregivers played a crucial role in determining the type of care received by older adults, particularly for individuals with varying levels of dependency [52, 83, 99], and caregivers living with older adults were more likely to be involved in care decision making than those who were not [100].

On the other hand, network size was not significantly associated with access to more support [39, 43]. The absence of informal care support was found to have a negative association with older adults’ health behaviour, including their tendency to seek treatment, engage in care, and adopt health-promoting behaviours [39, 72, 101]. Stigma was found to create barriers to developing strong social ties with their networks, particularly for older adults with low socio-economic status, cognitive impairment and physical disability [76, 79, 102].

The second theme focused on social engagement and network diversity. Our analysis revealed that active social and community engagement had a positive association with various aspects of older adults’ well-being, including mental well-being, quality of life, and life satisfaction [48, 64, 74, 77–82, 103]. Creating social space and opportunities was reported to have a positive effect on older adults’ social engagement [45, 71–78]. Social engagement was related to lower prevalence of cognitive impairment and incident dementia [80, 102, 104, 105]. In contrast, concerns have been raised in both qualitative and quantitative studies that cognitive impairment and activities of daily living (ADL) limitations created barriers for older adults to engage in community activities [45, 56, 64, 71, 74, 76, 80, 82].

The third theme focused on the utilization of formal care services. Our findings revealed that poor health status, advanced age, and an increased care burden were associated with a higher utilization of formal care services [50, 57, 58, 76, 84–89, 94, 100, 106–109]. In particular, ADL limitations and poor health status were found to be associated with an increase in care burden, which in turn was associated with more frequent healthcare use [30, 99, 100, 107, 108, 110]. Older adults with higher socio-economic status demonstrated a greater likelihood of utilizing care services provided by professionals or private care, in comparison to those with lower socio-economic status [56, 58, 83, 111, 112]. On the other hand, older adults with low socio-economic status were more likely to receive support from neighbourhood, publicly-funded care services and government financial support [40, 49, 54, 58, 83, 108, 111]. Findings regarding the relationship between socio-economic status and the utilization of formal care support were consistently observed across studies conducted in various countries and continents, including Asia, Europe, and America. The significant correlation between a longer duration of relationship and reduced use of healthcare services was reported by one quantitative study [88].

Taken together, as depicted in Fig. 3, the three theme groups exhibited close proximity to one another. Stronger social engagement was found to be associated with increased access to formal care services, attributed to the information and support acquired from community groups [55, 66, 75, 76, 86, 113]. Those receiving formal care continued to rely on informal care support [43, 53]. The extent to which older adults received informal care support was contingent upon the strength of their relationships within their social networks [31, 39, 46, 79].

### Risk of bias

Of the 34 qualitative studies (Supplementary Figure S1), 24 were rated as low risk of bias [28, 35, 38, 40, 41, 43, 45, 48, 49, 53, 55, 56, 64, 71, 73–76, 79, 92, 99, 107, 114, 115], nine as moderate risk of bias [37, 42, 44, 47, 54, 63, 72, 77, 108] and one as high risk of bias [46]. Of the 83 quantitative studies (Supplementary Figure S2-S3), 18 were rated as low risk of bias [3, 62, 70, 80, 86, 89, 96, 105, 113, 116–125], 56 as moderate risk of bias [7, 30, 31, 33, 34, 39, 51, 57–60, 66–69, 81–84, 87, 88, 90, 93, 94, 97, 98, 100–103, 109–112, 126–144] and nine as high risk of bias [36, 50, 65, 71, 85, 91, 106, 145, 146]. In respect to the four mixed-methods studies (Supplementary Figure S4), one had low risk of bias [52] and three had moderate risk of bias [29, 32, 78]. In sensitivity analysis (removal of studies with high risk of bias one by one to examine its influence on the results), no differences in the results were identified.

## Discussion

One hundred twenty-one studies were included in this systematic review. Of the studies examining social networks, we found that older adults in care were integrated into diverse networks comprising various groups, including both family and non-family members, which yielded reciprocal benefits. These benefits encompassed improvements in the wellbeing of older adults and a reduction in healthcare utilization. The presence of reciprocity and frequent contact emerged as positive factors associated with stronger relationships between older adults and their social networks. Moreover, in comparison to older adults residing far from their networks, those who cohabited or lived in close proximity to their networks were more likely to receive informal care support. Communication technology served as a valuable means of connecting older adults with their social networks and maintaining relationships, particularly when geographical distance separated them from their family members. Regarding formal care provision, increased healthcare use and decreased social/community engagement among older adults were associated with factors such as limitations in activities of daily living (ADL), advanced age, and poor health status. The lack of social/community engagement was found to have a negative impact on the mental wellbeing of older adults. On the other hand, the creation of social spaces and opportunities showed a positive correlation with older adults’ social engagement. It is noteworthy that the introduction of formal care support did not diminish the important role that informal care networks played in the lives of older adults.

Most prior reviews provided a narrow focus related to older adults’ social networks. The most relevant one was the systematic review by Siette et al. (2021) which synthesised studies that measured the social networks of older adults, specifically focusing on studies that did not have a primary focus on care [133]. Their results showed that the most commonly used dimensions in the measurement of social networks included the number of ties, content of networks (such as family and friends), contact frequency, social participation, social support, social satisfaction, and emotional bond. This review identified additional dimensions, including the intensity of networks, diversity of networks, reciprocity, and duration of relationships (Table S4). Environmental factors (such as proximity/location and opportunities for social events) were reported by two systematic reviews as important factors affecting older adults’ social participation [13, 147]. Similarly, we found that both the neighbourhood environment and the health conditions of older adults significantly influenced their social engagement. However, we also observed that the use of communication technology played a role in mitigating the impact of distance by connecting older adults with their social networks, even if they were living far apart. This finding resonates with four prior systematic reviews which showed that the use of social networking sites and communication technologies were positively associated with enhanced social participation and overall wellbeing among older adults [148–151]. In particular, the COVID-19 pandemic has significantly impacted the delivery and availability of both informal and formal care, highlighting the potential role of technology in promoting interactions and enhancing network diversity.

The strengths of this review include a transparent and rigorous synthesis of relevant evidence, along with a comprehensive and systematic examination of the concepts influencing the social networks of older adults and their associations. Despite the strengths of this review, there remain some limitations. First, the heterogeneity in study design precluded the possibility of conducting a meta-analysis to pool the quantitative results. However, to compensate for this, we employed network analysis and diagrams to identify patterns and density of the links between concepts as reported by the included studies. The second limitation lies in the fact that we did not weigh in the study design of included studies when synthesizing the quantitative and qualitative results. Given that narrative synthesis was the primary analytical approach employed in this review, we did not deem it advantageous to segregate quantitative findings from qualitative findings, particularly due to the substantial heterogeneity observed in the quantitative data. Instead, we treated all study findings as textual information and quantified the connections between concepts derived from these findings. Consequently, the analysis carried out in this study is exploratory in nature. Third, due to the broad research question addressed in this review, there is a possibility that the search strategy may have missed certain relevant studies.

This study has several implications for research, practice and policy. Firstly, the existing body of research on social networks in older adults often exhibits heterogeneity in terms of the types of networks studied. To address this, future research can adopt a more comprehensive and systematic approach to understanding social networks in older adults. This can involve developing a standardised social network measure to examining various types of networks including informal and formal networks. By exploring the different dimensions of social networks, researchers can gain a more nuanced understanding of the social support systems available to older adults and how they interact. Secondly, social support, engagement, and participation in various networks are crucial factors that can significantly influence an individual’s health and well-being. This highlights the importance of including social support and network engagement as integral components of care plans developed by formal care providers. Additionally, informal networks, such as family, friends, and community groups, should be acknowledged and included as significant contributors to a person’s care and support system. Thirdly, in the context of care planning for older adults, recognizing and incorporating the diversity of social networks is crucial for comprehensively addressing care needs. Care providers should consider assessing various sources of social network support to contextualise care planning. This could include identifying key individuals in their network, evaluating the quality and availability of support and understanding the roles that different network members play in the older adults’ life. Care providers can also consider cultural factors that may influence the composition and dynamics of the social networks. By recognizing and accounting for the diversity of social networks, care plans can be more holistic and person-centred. Fourthly, formal care can play a vital role in helping individuals who live far away from family and friends to make connections with the community. By actively assisting individuals in making connections with the community, formal care providers may help combat social isolation, enhance well-being, and improve the overall quality of life for older adults with immediate family nearby. Finally, Governments should promote and enable collaboration between informal and formal care providers. To encourage such collaborations and ensure caregivers to have the necessary tools and information for high quality care, policies could involve creating information-exchange systems, support networks and educational programmes. These could enable formal and informal caregivers to work together more closely and effectively.

To conclude, this study comprehensively investigated formal and informal care of older adults’ social networks and found that older adults were embedded within a diverse network. Policy and future research ought to prioritize and support the diversity in care. It is crucial to develop care plans that not only cater to the specific circumstances of older adults but also aim to foster strong bonds within their social networks, both within the community and through effective care coordination.

### Electronic supplementary material

Below is the link to the electronic supplementary material.

Supplementary Material 1

## Data Availability

All data generated or analysed during this study are included in the supplementary appendix.

## Abbreviations

LMIC: low- and middle-income countries
MeSH: medical subject heading
CASP: critical appraisal skills programme
MMAT: mixed-methods appraisal tool
LGBT: lesbian, gay, bisexual, and transgender
ADL: activities of daily living
