# Supplementary material for: Examining the social networks of older adults receiving informal or formal care: a systematic review
Source: BMC Geriatr. 2023 Aug 31;23:531. doi: 10.1186/s12877-023-04190-9 (PMC10470175; doi:10.1186/s12877-023-04190-9)
Supplement: Supplementary file 1 — Supplementary Material 1 [file 12877_2023_4190_MOESM1_ESM.docx]

Appendix: Supplementary tables and figures

Table of Contents

[Table S1: PRISMA reporting checklist 3](#_Toc130047026)

[Table S2: Search strategy 6](#_Toc130047027)

[Box S1: Further information on the analyses 8](#_Toc130047028)

[Table S3: Study characteristics 9](#_Toc130047029)

[Table S4: Definition of network-related concepts 36](#_Toc130047030)

[Table S5: Centrality score of network-related concepts in descending order 37](#_Toc130047031)

[Table S6: Links between concepts identified by included studies 39](#_Toc130047032)

[Table S7: Lists of concepts within the three communities 51](#_Toc130047033)

[Figure S1: Risk of bias assessment for qualitative studies 52](#_Toc130047034)

[Figure S2: Risk of bias assessment for cross-sectional studies 52](#_Toc130047035)

[Figure S3: Risk of bias assessment for cohort studies 53](#_Toc130047036)

[Figure S4: Risk of bias assessment for mixed-methods studies 53](#_Toc130047037)

[Table S8: Networks of older adults stratified by population and continent 54](#_Toc130047038)

[References 57](#_Toc130047039)

# Table S1: PRISMA reporting checklist

| **Section and topic** | **Checklist item** | **Location where item is reported** |
| --- | --- | --- |
| **Title** |  |  |
| Title | Identify the report as a systematic review | Main document (P1) |
| **Abstract** |  |  |
| Abstract | Follow PRISMA 2020 for abstract checklist | Main document (P2) |
| **Introduction** |  |  |
| Rationale | Describe the rationale for the review in the context of existing knowledge | Main document (P3-4) |
| Objectives | Provide an explicit statement of the objective(s) or question(s) the review addresses | Main document (P3-4) |
| **Methods** |  |  |
| Eligibility criteria | Specify the inclusion and exclusion criteria for the review and how studies were grouped for the syntheses. | Main document (P5) |
| Information sources | Specify all databases, registers, websites, organisations, reference lists and other sources searched or consulted to identify studies. Specify the date when each source was last searched or consulted | Main document (P4) |
| Search strategy | Present the full search strategies for all databases, registers and websites, including any filters and limits used | Appendix Table S2 |
| Selection process | Specify the methods used to decide whether a study met the inclusion criteria of the review, including how many reviewers screened each record and each report retrieved, whether they worked independently, and if applicable, details of automation tools  used in the process | Main document (P5-6) and Figure 1 |
| Data collection process | Specify the methods used to collect data from reports, including how many reviewers collected data from each report, whether  they worked independently, any processes for obtaining or confirming data from study investigators, and if applicable, details of  automation tools used in the process | Main document (P6) |
| Data items | List and define all outcomes for which data were sought. Specify whether all results that were compatible with each outcome  domain in each study were sought (e.g. for all measures, time points, analyses), and if not, the methods used to decide which  results to collect | Main document (P6) and Supplementary Table S4 |
|  | List and define all other variables for which data were sought (e.g. participant and intervention characteristics, funding sources).  Describe any assumptions made about any missing or unclear information | Main document (P6) and Supplementary Table S4 |
| Study risk of bias  assessment | Specify the methods used to assess risk of bias in the included studies, including details of the tool(s) used, how many reviewers  assessed each study and whether they worked independently, and if applicable, details of automation tools used in the process | Main document (P6-7) |
| Effect measures | Specify for each outcome the effect measure(s) (e.g. risk ratio, mean difference) used in the synthesis or presentation of results | Main document (P7-8) |
| Synthesis methods | Describe the processes used to decide which studies were eligible for each synthesis (e.g. tabulating the study intervention  characteristics and comparing against the planned groups for each synthesis) | Main document (P7-8) |
|  | Describe any methods required to prepare the data for presentation or synthesis, such as handling of missing summary statistics, or data conversions | Main document (P7-8) |
|  | Describe any methods used to tabulate or visually display results of individual studies and syntheses | Main document (P7-8) |
|  | Describe any methods used to synthesise results and provide a rationale for the choice(s). If meta-analysis was performed,  describe the model(s), method(s) to identify the presence and extent of statistical heterogeneity, and software package(s) used | Main document (P7-8) |
|  | Describe any methods used to explore possible causes of heterogeneity among study results (e.g. subgroup analysis, meta-regression) | Main document (P7-8) |
|  | Describe any sensitivity analyses conducted to assess robustness of the synthesised results | Main document (P7-8) |
| Reporting bias assessment | Describe any methods used to assess risk of bias due to missing results in a synthesis (arising from reporting biases) | Main document (P6-7) |
| Certainty assessment | Describe any methods used to assess certainty (or confidence) in the body of evidence for an outcome | Not applicable |
| **Results** |  |  |
| Study selection | Describe the results of the search and selection process, from the number of records identified in the search to the number of  studies included in the review, ideally using a flow diagram | Figure 1 |
|  | Cite studies that might appear to meet the inclusion criteria, but which were excluded, and explain why they were excluded | Figure 1 |
| Study characteristics | Cite each included study and present its characteristics | Main document (P9-10) and Supplementary Table S3 |
| Risk of bias in studies | Present assessments of risk of bias for each included study | Supplementary Figure S1-S4 |
| Results of individual  studies | For all outcomes, present, for each study: (a) summary statistics for each group (where appropriate) and (b) an effect estimate and  its precision (e.g. confidence/credible interval), ideally using structured tables or plots | Main document (P9-10) |
|  | For each synthesis, briefly summarise the characteristics and risk of bias among contributing studies | Main document (P9-10, 15 and Table 1) |
|  | Present results of all statistical syntheses conducted. If meta-analysis was done, present for each the summary estimate and its  precision (e.g. confidence/credible interval) and measures of statistical heterogeneity. If comparing groups, describe the direction  of the effect | Not applicable |
|  | Present results of all investigations of possible causes of heterogeneity among study results | Main document (P9-15) |
|  | Present results of all sensitivity analyses conducted to assess the robustness of the synthesised results | Main document (P15) |
| Reporting biases | Present assessments of risk of bias due to missing results (arising from reporting biases) for each synthesis assessed | Not applicable |
| Certainty of evidence | Present assessments of certainty (or confidence) in the body of evidence for each outcome assessed | Not applicable |
| **Discussion** |  |  |
| Discussion | Provide a general interpretation of the results in the context of other evidence | Main document (P15-19) |
|  | Discuss any limitations of the evidence included in the review | Main document (P15-19) |
|  | Discuss any limitations of the review processes used | Main document (P15-19) |
|  | Discuss implications of the results for practice, policy, and future research | Main document (P15-19) |
| **Other information** |  |  |
| Registration and  protocol | Provide registration information for the review, including register name and registration number, or state that the review was not  registered | Main document (P4) |
|  | Indicate where the review protocol can be accessed, or state that a protocol was not prepared | Main document (P4) |
|  | Describe and explain any amendments to information provided at registration or in the protocol | Main document (P6) |
| Support | Describe sources of financial or non-financial support for the review, and the role of the funders or sponsors in the review | Title page |
| Competing interests | Declare any competing interests of review authors | Title page |
| Availability of data,  code, and other  materials | Report which of the following are publicly available and where they can be found: template data collection forms; data extracted  from included studies; data used for all analyses; analytic code; any other materials used in the review | Appendix |

# Table S2: Search strategy

| **Database** | **Search term** |
| --- | --- |
| CINAHL | S1: TI ( (Network* or social network* or socio-ecolog* or support network* or social interaction* or family network* or friend relationship* or friend network* or local community network* or neighbourhood network*). ) OR AB ( (Network* or social network* or socio-ecolog* or support network* or social interaction* or family network* or friend relationship* or friend network* or local community network* or neighbourhood network*). )  S2: TI ( older adult* or older person* or older people or elderly or later life or senior* ) OR AB ( older adult* or older person* or older people or elderly or later life or senior* )  S3: TI ( Care or home care or care home or long term care or domiciliary care or carer* or paid care* or unpaid care or formal care or informal care or nursing home or community care or assisted living or retirement village ) OR ( Care or home care or care home or long term care or domiciliary care or carer* or paid care* or unpaid care or formal care or informal care or nursing home or community care or assisted living or retirement village )  S4: S1 AND S2 AND S3 |
| Embase | 1. (Network* or social network* or socio-ecolog* or support network* or social interaction* or family network* or friend relationship* or friend network* or local community network* or neighbourhood network*).tw.  2. (older adult* or older person* or older people or elderly or later life or senior*).tw.  3. (Care or home care or care home or long term care or domiciliary care or carer* or paid care* or unpaid care or formal care or informal care or nursing home or community care or assisted living or retirement village).tw.  4. social network  5. 1 or 4  6. home care/ or hospice care/ or long term care/ or geriatric care  7. 3 or 6  8. 2 and 5 and 7 |
| Medline | 1. Social Interaction/ or Social Networking/ or Online Social Networking/ or Social Environment/  2. (Network* or social network* or socio-ecolog* or support network* or social interaction* or family network* or friend relationship* or friend network* or local community network* or neighbourhood network*).tw.  3. 1 or 2  4. (older adult* or older person* or older people or elderly or later life or senior*).tw.  5. "Hospice and Palliative Care Nursing"/ or Hospice Care/ or Long-Term Care/ or Uncompensated Care/ or Patient Care/ or Nursing Care/ or Primary Health Care/ or "Delivery of Health Care"/  6. (Care or home care or care home or long term care or domiciliary care or carer* or paid care* or unpaid care or formal care or informal care or nursing home or community care or assisted living or retirement village).tw.  7. 5 or 6  8. 3 and 4 and 7 |
| PsycINFO | 1. (Network* or social network* or socio-ecolog* or support network* or social interaction* or family network* or friend relationship* or friend network* or local community network* or neighbourhood network*).tw.  2. (older adult* or older person* or older people or elderly or later life or senior*).tw.  3. (Care or home care or care home or long term care or domiciliary care or carer* or paid care* or unpaid care or formal care or informal care or nursing home or community care or assisted living or retirement village).tw.  4. exp Elder Care/ or exp Home Care/ or exp Long Term Care/ or exp Health Care Services/  5. 3 or 4  6. Social Communication/ or Social Networks/ or Social Groups/ or Online Social Networks/  7. 1 or 6  8. exp Aging/ or exp Geriatric Patients/ or exp Geriatrics/  9. 2 or 8  10. 5 and 7 and 9 |
| Web of Science | 1. TI=(Network* or social network* or socio-ecolog* or support network* or social interaction* or family network* or friend relationship* or friend network* or local community network* or neighbourhood network*) OR AB=(Network* or social network* or socio-ecolog* or support network* or social interaction* or family network* or friend relationship* or friend network* or local community network* or neighbourhood network*)  2. TI=(older adult* or older person* or older people or elderly or later life or senior*) OR AB=(older adult* or older person* or older people or elderly or later life or senior*)  3. TI=(Care or home care or care home or long term care or domiciliary care or carer* or paid care* or unpaid care or formal care or informal care or nursing home or community care or assisted living or retirement village) OR AB=(Care or home care or care home or long term care or domiciliary care or carer* or paid care* or unpaid care or formal care or informal care or nursing home or community care or assisted living or retirement village)  4. 1 and 2 and 3 |
| Cochrane | 1. (Network* or social network* or socio-ecolog* or support network* or social interaction* or family network* or friend relationship* or friend network* or local community network* or neighbourhood network*):ti OR (Network* or social network* or socio-ecolog* or support network* or social interaction* or family network* or friend relationship* or friend network* or local community network* or neighbourhood network*):ab  2. (older adult* or older person* or older people or elderly or later life or senior*):ti OR (older adult* or older person* or older people or elderly or later life or senior*):ab  3. (Care or home care or care home or long term care or domiciliary care or carer* or paid care* or unpaid care or formal care or informal care or nursing home or community care or assisted living or retirement village):ti OR (Care or home care or care home or long term care or domiciliary care or carer* or paid care* or unpaid care or formal care or informal care or nursing home or community care or assisted living or retirement village):ab  4. 1 and 2 and 3 |

# Box S1: Further information on the analyses

Centrality analysis: Degree centrality and eigenvector centrality were computed to characterise the extent of ties and influence a concept has on a network [1]. A concept (node) with a high level of degree centrality indicates being linked with a larger number of other concepts, and one with a high level of eigenvector centrality indicates being linked with a larger number of influential concepts (that shared links with many concepts).

Louvain algorithm: The Louvain algorithm was chosen because it focuses on maximising within-community connections and minimising outside-community connections [2]. This method allowed us to identify which concepts were closely connected and formed a theme (community) distinct from other themes, and the number of communities (themes) within the network of concepts being studied. Modularity was used to measure the strength of community division. Value for modularity close to 1 indicates strong community structure but it usually falls in the range between 0.3-0.7 [3]. The package used for the analyses is ‘igraph’[4]. There is clear guidance on how to create a graph dataframe, calculate distance and plot figures in reference 4.

# Table S3: Study characteristics

| **Author** | **Country** | **Country income** | **Sample size** | **Type of research** | **Participant** | **Setting** | **Mean age** | **Analysis** | **Risk of bias** | **Results** |
| --- | --- | --- | --- | --- | --- | --- | --- | --- | --- | --- |
| Aida et al.(2016) [5] | Japan | High | 19756 | Quantitative | Older adults | Community | NA | Logistic regression | Low | Increased network diversity (adjusted OR: 1.06 95%CI=1.02-1.09) and network size (adjusted OR: 1.03 95%CI=1.00-1.05) was positively associated with oral health behaviour among older adults |
| Al-Kandari (2011)[6] | Kuwait | High | 1427 | Quantitative | Older adults | Community | 77 | Multivariate regression and T-test | Moderate | Strength of relationship, frequency of contact, age and living with children and spouse were significantly associated with somatic symptoms |
| Aung et al. (2020)[7] | Japan | High | 243 | Quantitative | Older adults | Community | 74 | Multivariate regression | High | 1. Engagement in community activities (e.g. voluntary or paid work and exercise programme) was significantly associated with the diversity of social networks, active ageing. 2. Internet access positively influenced elderly's diversity of social networks and prevented social isolation 3. Assistance needs achievable in the home settings was associated with quality of life. 4. Participation in group physical activities in leisure time, were statistically significant in relation to active ageing. 5. Needs for personal care or assistance were met in their home setting using formal services |
| Ayalon (2019)[8] | Israel | High | 229 | Quantitative | Older adults | Community | 85 | Latent profile analysis | Moderate | 1. Older adults with friend-based networks and child-based networks were indistinguishable in terms of their physical and mental well-being. 2. Individuals classified into the friends-based profile were significantly younger than those classified into the child-based profile. |
| Ayalon et al. (2013)[9] | Israel | High | 48 | Qualitative | Older adults | Community | NA | Thematic analysis | Low | 1. Three major themes were identified: a time dimension, representing the perceived qualities of relationships based on the time during which the relationships were first formed (the longer duration of relationships increased its quality); and two spatial dimensions, representing the CCRC vs. the community at large and private vs. public space and identity. 2. Relationships that were established in the community or that flexibly cross the dividing line between the community and the CCRC tend to be considered more intimate and meaningful. 3. Taking part in social events enhanced social relationships in the community. 4. Geographical distance influenced social ties. 5. Deterioration of physical health and mobility influenced social interactions. 6. Relationships that were formed many years ago were usually of lasting quality. |
| Ayalon et al (2019)[10] | Israel | High | 229 | Quantitative | Older adults | Community | 85 | Validation | High | The only consistent determinants of the presence of close social ties were the physical location indicators |
| Baltes et al. (1983)[11] | USA | High | 40 | Quantitative | Older adults | Care institution | 81 | Correlation tests | High | 1. Non-engaged behaviour (including in a social space) did not lead to reactions or acts of social partners. 2. Individual dependence (dependent behaviours) acted as prompts for social partners to behave in a congruent fashion and provided support for their dependence |
| Barros et al. (2008)[12] | Brazil | Upper-  Middle | 4 | Qualitative | Older adults | Community | NA | Case study | Moderate | 1. The interaction between elderly and their family influenced their active engagement in the care for elderly person. 2. Taking part in the social events and community activities affected elderly's connections with others. 3. Some attended the stomatherapy service regularly, contact with the other participants helped them to understand that they were not alone. |
| Bear (1990)[13] | USA | High | 81 | Quantitative | Older adults | Care institution | NA | Logistic regression | Moderate | 1. The lower the older adults' functional status, the less likely they would return home (at time 2). 2. Health status significantly affected the probability of returning home. 3. The higher the intensity of the elders' ties with their personal network at the time of their move into the care home, the lower is the likelihood of elders returning home |
| Berglund et al. (2016)[14] | Sweden | High | 179 | Quantitative | Older adults | Community | NA | Logistic regression | Low | Contacts with others besides children adds further to our understanding of circumstances contributing to high life satisfaction. On the other hand, contact with children had no significant impact. |
| Bijnsdorp et al. (2019)[15] | Netherlands | High | 146 | Quantitative | Older adults | Community | 81 | Latent class analysis | Moderate | A professional network (40%) was identified in which the older persons received care from home care staff. Nearly 40% of the care recipients in the professional network had a partner, but did not receive care from them. Care recipients in this network were relatively old. |
| Boneham et al. (2006)[16] | UK | High | 19 | Qualitative | Older adults with low SES | Community | 69 | Thematic analysis | Low | 1. Strength of relationship influenced their social networks (friends, children, spouse and neighbours) and health behaviour 2. Poor relationships with health professionals affected their health behaviour 3. Reciprocal family help was perceived by some as embarrassing. 4. Stigma related to drug use affected their social networks and the strength of relationships with others 5. Engagement in community events enhanced their mental health and wellbeing |
| Brennan-Ing et al. (2014)[17] | USA | High | 210 | Mixed methods | LGBT older adults | Community | 60 | Correlation tests and grounded theory | Low | 1. Two-thirds of participants lived alone.2. Women had significantly larger size of social networks, but they significantly had higher likelihood of having negative family support than men. 3. Friends were the most prevalent functional network element in this population (77%) 4. A number of respondents could not engage in community programmes due to their financial difficulties |
| Brito et al. (2018)[18] | Brazil | Upper-  Middle | 1413 | Quantitative | Older adults | Community | NA | Logistic regression | Moderate | 1. There was no statistically significant difference observed between the number of members in the network and level of dependency. 2. Dependent elderly people received more material support, whereas independent elderly people received more emotional support |
| Buckley et al. (2009)[19] | Ireland | High | 10 | Qualitative | Older adults | Care institution | 83 | Thematic analysis | Low | 1. Residents described their relationship with other residents as not very close, as they did not have the same mental ability. 2. Medical staff were able to understand them, which increased the connectedness |
| Canham et al. (2018)[20] | Canada | High | 24 | Qualitative | Older adults with low SES | Community | NA | Thematic analysis | Low | The effective use of the social environment within the physical environment can offer socially isolated seniors more opportunity to engage in community programmes and enhance connectedness |
| Carpenter (2002)[21] | USA | High | 32 | Quantitative | Older adults | Care institution | 67 | Multivariate analysis | Moderate | 1. Network size was not significantly associated with other social support variables (e.g. social competency or satisfaction). 2. None of the psychological measures were significantly related to support network size 3. Perceived support was significantly associated with psychological well-being, satisfaction and social competency 4. Patients with low motivation to participate in treatment reported less support from family and peers. 5. Peers and healthcare staff were significantly considered as functional networks than family |
| Coe et al. (1985)[22] | USA | High | 394 | Quantitative | Older adults | Community | NA | NA | High | 1. There was no significant difference in hospital utilisation between patients with family and without 2. Elderly without access to family support used emergency care services more frequently |
| Coe et al. (1984)[23] | USA | High | 401 | Quantitative | Older adults | Community | NA | One-way analysis of variance | Moderate | One trend is clear, namely, the disadvantaged status of older persons abandoned by both family and neighbour networks. They reported the poorest perceived health, the poorest mental health, the most limitation of activity |
| Cohen et al. (2017)[24] | USA | High | 16 | Qualitative | Older adults | Community | NA | Thematic analysis | Low | For elders with multiple needs, it is important that supporting tasks are distributed rather than falling on a single or primary supporter. When network members connect and/or work together to coordinate efforts, responsibility does not fall to a single family member. If family is too far to be of assistance, coordinating with neighbours is essential |
| Cott et al. (2008)[25] | Canada | High | 13 | Qualitative | Older adults | Community | NA | Thematic analysis | Low | The seniors reported seeing or talking with friends and family members more often than with health care professionals. |
| Crooks et al. (2008)[26] | USA | High | 2249 | Quantitative | Older adults | Community | 80.6 | Cox proportional hazards | Low | 1. Daily contact reduced the adjusted HRs for dementia by almost half (0.57; 95% CI=0.38, 0.87). 2. Satisfaction with amount of contact did not reduce the risk of dementia, with an adjusted HR of 0.70 (95% CI=0.48, 1.01). 3. Compared with women with smaller social networks, the adjusted hazard ratio for incident dementia in women with larger social networks was 0.74 (95% confidence interval=0.57, 0.97). |
| Dagnan et al. (1997)[27] | UK | High | 52 | Quantitative | Older adults | Care institution | 63.6 | Descriptive analysis | High | 1. The mean number in a social network was 3.1. 2. Older people had fewer contacts with their families (mean number of contacts: 46.7) compared to co-residents (mean number of contacts: 235.4) or friends with learning disability (mean number of contacts: 50.5) |
| Doekhie et al. (2020)[28] | Netherlands | High | 133 | Mixed methods | Older adults | Community | 81.1 | One-way analysis of variance, multinomial logistic regression analysis and content analysis | Low | The more social support a patient perceives themselves as having, the more the patient will perceive themselves as having a shared role in the decision-making process. |
| Doubova et al. (2010)[29] | Mexico | Upper-  Middle | 3348 | Quantitative | Older adults | Community | NA | Cluster analysis and poisson regression | Moderate | 1. The mean network size: 6.4. 2. Five types of social networks were identified: diverse network with community participation (12.1%, mean network size: 6.3), diverse network without community participation (44.3%, mean network size: 6.8); widowed (32.0%, mean network size: 6.8); nonfriends-restricted network (7.6%, mean network size: 7.3) and nonfamily-restricted network (4.0%, mean network size: 1.2) 3. Diverse network participation and the nonfriends-restricted network received instrumental support to perform BADL and/or IADL and economic support, more often than those embedded in the other networks. The nonfamily restricted was the group that received less instrumental and economic support. 4. Older adults with dependency in BADL-IADL more likely belonged to the widowed network (Adjusted Prevalence Ratio: 1.5; 95% CI: 1.1-2.1). |
| Drennan et al. (2008)[30] | Ireland | High | 683 | Quantitative | Older adults | Community | 73.5 | Chi-square | Moderate | 1. Overall the vast majority of respondents (73.2%) were in locally integrated support networks, indicating that they had close relationships with family friends, community, relatives and neighbours. |
| Duner et al. (2007)[31] | Sweden | High | 22 | Qualitative | Older adults | Community | NA | Open and focused coding | Low | 1. The mean formal network size was 7.1, and informal network size: 3.1. 2. The size of the formal support network varied from one to 12 people (or categories of people), and the size of the informal support network varied from one to six people (or categories of people). Those include children, relatives, friends, neighbours, and spouse. 3. The findings reflect the importance of informal support to older people, even those who also receive formal support. 4. The geographical distance between an older person and their family affected how often they met and how much help was given. When family members lived far away, the older person seldom met them and rarely received practical help and most contacts were through frequent telephone conversations. 5. The formal support network, i.e. social-care and health-care staﬀ, is obliged to provide help regardless of geographical distance. |
| Dupuis-Blanchard (2007)[32] | Canada | High | 20 | Qualitative | Older adults | Care institution | NA | Thematic analysis | Low | 1. This study provides a new understanding of the concept of social engagement. Factors influencing social engagement include environmental context (common area) and personal biological (physical disability), psychological (perceived connectedness) and social factors (strength of relationship). 2. Some actively took part in social activities and considered themselves active. If seniors lost their driving privileges, they maintained contact with family through telephone. |
| Ehrlich (1985)[33] | USA | High | 80 | Quantitative | Older adults | Community | NA | Correlation tests | High | 1. Formal services were used to a much greater extent by respondents in town than those in outlying areas.2. Proximity of residence was an important factor for network functionality. People in more rural, outlying areas who are highly functional remain in the community. 3. The reciprocal helping patterns partially account for the perception of independence. 4. Every respondent has a network system consisting of more than one category. 5. Aside from relationships with children, the networks were highly peer related. Spouses were the main helpers for those with intact marriages. Kin (children and relatives) assisted with more tasks and friends and neighbours ranked higher than kin in some, with at least monthly contact and assistance with basic IADL tasks. |
| Evans et al. (2018)[34] | Malaysia | Upper-  Middle | 40 | Qualitative | Older adults | Community | 75 | Thematic analysis | Low | All participants said they lived alone; living arrangements, however, were often complex. For Malays, most support came from nearby adult children and relatives, whereas Chinese participants, who less frequently had adult children living locally, emphasised support from friends and neighbours (including religion). Immigrant adult children’s assistance was mostly informational and ﬁnancial, instrumental assistance was either substituted for money, or provided solely during periods of ill-health. Physical decline, limited telephone use, inadequate transportation and fears of crime were barriers to social support. Some participants with the use of informal care relied on government financial support |
| Fernández‐Carro et al. (2019)[35] | Europe | High | 37708 | Quantitative | Older adults | Community | NA | Binomial regression | Moderate | 1. A larger network size was positively associated with the use of informal care 2. A negative association between poor health status and receiving informal care 3. Age decreased the probability of receiving informal care 4. Geographical proximity was significantly associated with the probability of receiving informal care (co-residing and living nearby increased the use of informal care). 5. As ADL limitations increased, the probability of using informal care decreased but using both informal and formal (combined) care increased. 6. Maintaining daily contact enhanced network bonding |
| Gallo (1984)[36] | USA | High | 192 | Quantitative | Older adults | Community | 70.3 | Multiple regression | Moderate | 1. The larger a network, the higher a respondent's health status and the lower his or her-utilization of health care services. 2. The percentage of contacts with family members was negatively associated with healthcare use but was not statistically significantly associated with health status. 3. Networks composed of members with similar demographic traits and networks in which members were close to one another tended to be associated with high health status and the low utilization of health care services. 4. Member-initiated networks (shy personality) were associated with respondents' low health status and their high utilization of health care services. 5. Long distance between the respondent and members of the network was associated with high health status and the low utilization of health care services. 6. The longer the duration of a relationship, the lower the utilization of health care services but was not associated with health status. 7. The extremely weak relationship of frequency of contact to either health status or the utilization of health care services. |
| Giles et al. (2007)[37] | Australia | High | 1477 | Quantitative | Older adults | Community | 79.8 | Multinomial logistic regression | Moderate | 1. Higher scores for confidant networks (i.e. those with children, other relatives, friends, confidants, and total social networks) appeared protective against nursing home use (odds ratio [OR] upper versus lower tertile of confidant networks = 0.50; 95%CI 0.33–0.75). |
| Golden et al. (2009)[38] | Ireland | High | 1334 | Quantitative | Older adults | Community | NA | Clustering | Low | 1. Social engagement (the frequency of attendance at religious and non-religious community events, and social interaction – the frequency of contact with neighbours and friends) was associated with a lower age- and sex-adjusted prevalence of depression (odds ratio for a one-tertile increase 0.48), generalised anxiety disorder (OR 0.60), cognitive impairment (OR 0.68) and physical disability (OR 0.62) all p > 0.001. 2. Adjusted for age, sex, depression, cognitive impairment and disability, the social engagement domain was also associated with better quality of life (OR 1.5) self-rated happiness (OR 1.3) and rating life as worth living (OR 1.4). 3. The family support dimension – proximity of kin and frequency of contact – was not significantly associated with any health outcome. |
| Golden et al. (2009)[39] | Ireland | High | 1299 | Quantitative | Older adults | Community | NA | Logistic regression | Moderate | 1. Having a non-integrated social network (family dependent) was associated with a decreased odds of being very happy (OR:0.75, p:0.040) and being satisfied with one’s life (OR:0.53, p<0.001). 2. Non-integrated social network was associated with 81% of the risk of depression in those with one or both factors, and with 70% of the overall risk of depression in the elderly population. |
| Greaves et al. (2006)[40] | UK | High | 172 | Mixed methods | Older adults | Community | 77 | Thematic analysis/two-sided t test | Moderate | 1. Social engagement interventions significantly increased mental component scores in SF12 at 6 months and 12 months. 2. Availability of activities and transport issues were frequently reported barriers to attending activities. For those who were engaged at 6 months, ill health was the main reason for non-engagement |
| Green (2016)[41] | UK | High | 16955 | Quantitative | LGBT older adults | Community | NA | Descriptive analysis | Moderate | 1. Almost a third of older LGBT adults never see kin. The prevalence of childlessness amongst older LGBT adults only partly explains this. These individuals also report that they never see their mother or father either because they are not alive or otherwise not in contact. 2. Of those who do interact, older heterosexuals are far more likely to do this at least weekly (amalgamating “at least once per week” and “daily”) at 40.2 per cent of the sample compared to 30.7 per cent amongst older LGBT adults. 3. A greater proportion of older LGBT adults (63.8 per cent) mention that relatives are too far away to visit compared with 56.9 per cent of older heterosexuals and this difference is statistically significant. 4. Friends of older LGBT adults are more likely to provide informal care owing to the lack of kin contacts. |
| Gu et al. (2017)[42] | China | Upper-  Middle | 4293 | Quantitative | Older adults | Community | 77.5 | Multilevel multinomial analyses | Moderate | 1. Child caregivers were undoubtedly the dominant category, although they showed a sharply decreasing trend from approximately 62% in 2002 to less than 50% in 2008. spousal caregivers were the second important type, with dramatic growth from less than 25% in 2002 to almost 40% in 2011. Other caregivers, mainly including social service workers and housekeepers, saw a gradually decreasing trend from approximately 11% in 2002 to approximately 7% in 2011. 2. Compared to urban elderly individuals, rural ones are nearly twice as likely to have both spousal and child caregivers. 3. elderly individuals from the higher socio-economic classes being less likely to have both spousal caregivers and child caregivers compared to elderly individuals from lower socio-economic classes who have a greater chance of being cared for by spouses only. |
| Jacobs et al. (2018)[43] | Netherlands | High | 491 | Quantitative | Older adults | Community | 78.4 | Latent class analysis and multinomial regression analyses | Moderate | 1. The first network type is the privately paid network (n = 138, 28%). The older adults in this network type mostly received household care. They can be characterized as being in relatively good health, with many financial and social resources, and in control of their lives and their care. 2. The second network with predominantly co-residential care was distinguished (n = 78, 16%). Older adults co-residing informal caregivers, were always present in this network type, sometimes in the co-presence of privately paid help (in 18% of cases). 3. A large and more diverse informal care network was identified (n = 105, 21%). Older adults in this network had help from non-co-residing children (in 71% of cases), other family (23%) and neighbours (15%). 4. Publicly paid care network was distinguished (n = 170, 35%). All older adults in this network type received publicly paid help. 5. Older adults with a co-residential and publicly paid care network were in relatively poor health and that those with a privately paid care network were in relatively good health. 6. Many older adults had relatives, neighbours, and friends living close by, but these types of relationships were only marginally involved in the care network. |
| Jacobs et al. (2016)[44] | Netherlands | High | 74 | Quantitative | Older adults | Community | 82.8 | Multi-level logistic regression | Moderate | 1. When the informal care-giver was residing with the care recipient, the likelihood of discussing the care between a formal and an informal care-giver was higher (probability = 0.46) than for other informal care-givers (0.17). 2. When a care-giver performed more types of tasks instead of one, the likelihood that the informal and formal care-giver discussed the care was greater (probability = 0.38 versus 0.22). |
| Kuiper et al. (2020)[45] | Netherlands | High | 378 | Quantitative | Older adults | Community | 70.7 | Linear regression | Low | 1. Neither social network size nor loneliness was associated with cognitive impairments or decline. |
| Lee (2004)[46] | Hong Kong | High | 50 | Qualitative | Older adults | Community | NA | Thematic analysis | Moderate | 1. Elderly living with children, in particularly living with daughters, seemed to feel they receive better daily care support than those not living with children. 2. Geographical distance is an important factor in the provision of daily care support. Elderly living with children or with adult children living nearby often feel a sense of security and often indicated that they like to be sure they can find immediate help from adult children. 3. Elderly, in particular those from the lower socioeconomic group, noted they preferred to depend on the government financially, and wished that their adult children provided top-up pocket money for them to pursue leisure activities if they could afford to. 4. Elderly who are living with their adult children appeared to receive better emotional support than those living without children. 5. Those with low socio-economic status required government financial support |
| Loeb et al. (2003)[47] | USA | High | 37 | Qualitative | Older adults | Community | 72 | Thematic and content analysis | Low | 1. Key strategies identified were relating with health care providers, medicating, exercising, changing dietary patterns, seeking information, relying on spirituality and/or religion, family visits and engaging in life. Although social support was not mentioned as a discrete strategy, the participants’ social networks were embedded in all of the categories. 2. When family was unavailable other support networks emerged. Neighbours often stepped in to help with chores and activities. 3. Spousal social support was particularly evident in participants’ stories regarding dietary management. 4. When a spouse was not in the picture others rose to assume this supportive role. Children were prominent support partners. 5. Friendship networks often centred on regular group activities |
| Li et al. (2004)[48] | USA | High | 200 | Quantitative | Older adults | Community | 81 | Logistic regression | Moderate | 1. Only 2 of 202 elderly persons had no informal caregivers. All others had at least one informal caregiver, and 7% had four informal caregivers in their network. The average size of the informal network was 1.85. 2. Among the characteristics of informal caregiving networks, informal caregivers’ decision-making ability was a significant factor related to the probability of using formal services. The odds ratio was 0.53 (95% confidence interval: 0.29–0.96). 3. Other characteristics of informal caregiving networks, such as levels of assistance, size of informal networks, relationship between caregivers and care receivers, and emotional closeness between caregivers and care receivers were not significantly related to formal service use. 4. Compared with their counterparts, elderly persons who were Medicaid recipients (p < .0001), with an annual income equal to or higher than $10,000 (p = .03), and with higher levels of functional dependency (p = .05), were more likely to use formal services. |
| Li et al. (2021)[49] | China | Upper-  Middle | 3157 | Quantitative | Older adults | Community | NA | Logistic regression | Moderate | 1. Older adults on average had 3.25 (SD ± 1.49) network members with strong ties. A majority (95.2%) of network members were kin. 2. Both network size and volume of contact in the quantitative dimension were significantly associated with depressive symptoms. 3. However, the relationship between the volume of contact and depressive symptoms became non-significant after the qualitative dimension (i.e. emotional closeness) was added in a model. In other words, the significant relationship between volume of contact and depressive symptoms could be explained by emotional closeness. 4. A larger network size, higher proportion co-residents, and higher emotional closeness were associated with lower depressive symptoms. |
| Litwin (1998)[50] | Israel | High | 140 | Quantitative | Older adults | Care institution | NA | Multivariate analysis | Moderate | 1. The more frequent the contact with the network (friends, neighbours and family), the greater the provision of support by elderly respondents. 2. Residents in voluntary or private facilities reported more support provision than did their counterparts in public facilities. 3. The greater the perceived availability of support, the greater the reported provision of support. 4. As respondents' age increased, the amount of support reported to be provided by them decreased. |
| Litwin (1999)[51] | Israel | High | 2646 | Quantitative | Older adults | Community | 72.3 | Multivariate analysis | Moderate | 1. The attenuated network also had the highest relative degree of persons who neither provided nor received support of any kind. 2. Elders in the diversified network made significantly less use of formal services than those in all the other network types. 3. On the other hand, those in the religious family focused and the traditional extended family networks made greater than average use. 4. The friend and neighbour, narrow family focused and attenuated networks, on the other hand, each approached the average rate of utilization 5. The old-old made greater than average use of paid assistance than the young-old, as did women and those with some ADL disability. |
| Liu et al. (2017)[52] | UK | High | 44 | Qualitative | Older adults | Community | NA | Grounded theory | Low | 1. To maintain their quality of life and to adapt to the social environment of the UK, these Chinese immigrants addressed their problems mainly by seeking support from, and accessing services through other people. These people were described in this study as ‘Bridge People’. 2. For example, those who lived with their children had direct and frequent contact with them, which enhanced timely support with translation; otherwise they would have to wait until they contacted them. 3. Friends with better English proficiency could be contacted by telephone, during weekly social events or at a meeting point; and professional interpreters were always booked when arranging service appointments. 4. Getting help from Bridge People did not cost the participants any money. Professional interpreters are paid for by NHS or social care departments. Volunteers from family, social networks or NGOs provided help without charge. |
| McDonald et al. (2008)[53] | Australia | High | 6 | Qualitative | Older adults | Community | NA | Thematic analysis | Moderate | The central theme emerging from the data was support. Friendship was seen as being essential for older adults. The text offered examples of the participants’ essence of life, which was expressed as feelings of being needed and a strong sense of belonging. The text also offered examples of learning to adapt, expressed by the participants as the ability to gain confidence within themselves and share with each other in such a way as to meet each other’s needs. |
| McFarland (1999)[54] | USA | High | 107 | Quantitative | Older adults | Community | NA | One-tail t test | High | 1. When home health services were provided to elderly, informal support networks spent less time providing instrumental tasks for them. However, elderly who had high functional limitations did not experience a significant decrease in the time the informal support system provided instrumental tasks after the formal services were introduced. This is also true for those in the older age range (81 or older). |
| McLeod et al. (2008)[55] | UK | High | 17 | Qualitative | Older adults | Community | NA | Thematic analysis | Low | 1. Voluntary social care sectors enabled older service users to engage in social contacts outside their home, access to material and social resources, receive educational support, and connect with specialized health care providers. |
| Neves et al. (2019)[56] | Canada | High | 12 | Qualitative | Older adults | Care institution | 82 | Thematic analysis | Low | 1. The app increased sense of social interaction (communication frequency and type) with family and friends. 2. The app allowed these four participants to reconnect, communicate more often, and deepen relationships with relatives living abroad or afar. |
| Rodríguez et al. (2018)[57] | Austria, Germany, Sweden, Netherland, Spain, Italy, France, Denmark, Belgium, Czech Republic, Poland, Hungary, Portugal, Slovenia and Estonia | High | 17284 | Quantitative | Older adults | Community | NA | Logistic regression | Moderate | 1. The likelihood of receiving informal care increases with age and, especially, with the degree of dependency. 2. The likelihood of receiving informal care increases when there are children in social network. 3. The shorter the distance between the dependent older person and the members of his/her social network, the higher the likelihood of receiving informal care (OR 1.13, CI 1.04–1.25 in the first model and OR 1.11, CI 1.01–1.23 in the second one for a distance to members of the social network between 1 and 5 km). 4. When there is no close social network the likelihood of receiving informal care substantially decreases (OR 0.48, CI 0.40–0.56 in the first model and OR 0.46, CI 0.38–0.54 in the second one). 5. The key role played by children in social networks is confirmed by their high presence in the social network of those receiving informal care (65%). |
| Moorman et al. (2008)[58] | USA | High | 1968 | Quantitative | Older adults | Community | 69.6 | Logistic regression | Moderate | 1. In the process of choosing an end-of-life surrogate, most married people prefer a spouse and most unmarried parents prefer an adult child. 2. Low-quality next-of-kin relationships pushed older adults away from next-of-kin surrogates. 3. Those who bypassed their children reported significantly less family support (OR = 0.61, p < .05) and larger networks (OR = 1.39, p < .001) characterized by greater average closeness (OR = 2.15, p < .001) than those who chose a child |
| Nielson et al. (2019)[59] | New Zealand | High | 12 | Qualitative | Older adults | Community | NA | Thematic analysis | Low | 1. Living in a retirement complex, residents negotiated a social space to establish social groups. 2. If a resident experiences health decline and can no longer live independently, s/he relocates to a different accommodation where s/he is able to receive care, such as assisted living, the rest home, or hospital. 3. Social occasions were difficult for residents who started showing signs of cognitive decline, which impacted their ability to participate in conversation and was a prerequisite to group membership, and also for those with physical limitations requiring the use of mobility walkers or wheelchairs. 4. These experiences illuminate the problems older people have, such as isolation, exclusion and stigma, when they lose their independence. 5. As they got older, it was harder for them to make friends |
| Oh et al. (2019)[60] | USA | High | 4712 | Quantitative | Older adults | Community | 78 | Fine & Gray competing risks hazards model | Low | 1. The lack of social support (no social network, individuals who live alone, and lack of participation in social activities) were strong predictors of transitioning out of the home and into an institution. 2. Participants who had no social network (zero people in network) had a higher probability of institutionalization compared to participants with three or more people in their social network (adjusted sHR=1.8, 95% CI 1.2–2.5). 3. Participation in certain social activities was a strong predictor of delaying the transition out of the home and into an institution. 4. Participants over 80 years; white participants, participants with possible or probable dementia, functional disability (requiring help with one to two ADLs), and with two or more coexisting conditions, were institutionalized at higher rates compared to participants younger than 80 years, Black and Hispanic participants, participants with no dementia, no functional disability, and zero or one coexisting condition. |
| Vos-den Ouden et al. (2021)[61] | Netherlands | High | 16 | Qualitative | Older adults | Community | NA | Grounded theory | Low | 1. Due to health decline, older adults had less social lives and contacts. 2. Respondents were isolated from friends and had to deal with a cooled off contact with biological family members |
| Park et al. (2016)[62] | South Korea | High | 2089 | Quantitative | Older adults | Community | NA | Clustering and regression | Moderate | 1. Members in the restricted type (18%) were least connected with friends. Individuals in couple-focused networks (32%) had similarly limited social connection. Those in the friend network type (35%) had the highest contact with friends. The diverse network type had the most extensive social ties. 2. Network types that have more social resources do not play a significant role on checkups or hospitalizations. 3. There was no significant main effect of the friend network type on any health-care use measures |
| Peek et al. (1997)[63] | USA | High | 5151 | Quantitative | Older adults | Community | NA | Descriptive analysis | High | 1. The proportion of respondents that received no help from others, however, did diminish as the sample aged. Among those who did receive some form of help, the most common care configuration was composed exclusively of informal helpers. 2. As the sample aged, the proportions receiving only formal help and mixed help rose steadily. 3. Community dwelling elders receiving mixed helping networks had poorer health status |
| Pleschberger et al. (2017)[64] | Austria | High | 15 | Qualitative | Older adults | Community | 62.8 | Thematic analysis | Low | 1. As physical care needs increased, formal caregivers were introduced. Many neighbours drew the line, whereas friends seem to be more agreeable to take on physical care activities. |
| Powers (1988)[65] | USA | High | 69 | Qualitative | Older adults | Care institution | NA | Content analysis | Low | 1. Some terms used to express ideas about status support are social integration and participation. 2. Location was a factor in description of interactions among residents.3. The relationships tended to be more intense in the common areas.4. Statements were articulated by elderly residents concerning the importance of recognition, acceptance, love and affection, status, giving receiving in conferring a sense of happiness and wellbeing. The network interactions show the greater instances of instrumental and reciprocal resource exchange. 5. A greater number of tie does not necessarily indicate access to more support. 6. Institutionalized residents continued to depend on outside ties (family and friends) to meet everyday material and emotional needs. 7. Some residents found comfort in regular visits from their cat. |
| Powers (1996)[66] | USA | High | 69 | Qualitative | Older adults | Care institution | 73 | Thematic analysis | Moderate | 1. The network ties varied in terms of emotional intensity and reciprocity. 2. Physical proximity created opportunities for companionship and helpful exchanges. 3. Some residents interacted with others by sharing food and feeding a bird together |
| Powers (1992)[67] | USA | High | 69 | Qualitative | Older adults | Care institution | 73 | Thematic analysis | Moderate | 1. Relationships with staff were seen as useful in a political sense to ensure one's voice was heard but not for intimacy. 2. Friendship was distinguished from staff partnership on the basis of emotional closeness. 3. The frequency and intensity of relationships with staff were low |
| Prosser et al. (2008)[68] | Australia | High | NA | Qualitative | Older adults | Care institution | NA | Thematic analysis | Moderate | 1. The programme enhanced residents' mental well-being. 2. The programme has prompted social interactions with animals and other residents. |
| Reed (2006)[69] | USA | High | 82 | Quantitative | Older adults | Care institution | NA | Regression | Moderate | 1. 99%, identified having some network members who were family / kin other than offspring. 66% having a sibling among their social networks. 2. The average network size was 11. 3. Better health is positively associated with satisfaction with amount of received informational support and emotional support. 4. the better one’s health, the more likely they were to have a sense of social integration or to have friends who lived in the community rather than only friends living in the assisted living facility. 5. The respondents preferred relationships that were characterized by exchanges no matter what their health or how much support they received. |
| Rennemark et al. (1999)[70] | Sweden | High | 184 | Quantitative | Older adults | Community | NA | Logistic regression | Low | 1. In the female group, good subjective health correlated with satisfying social network structures and functions. 2. In men, there was no overall correlation between the symptom report and social network score (counting the number of social activities and contacts with friends, neighbours and children). 3. Satisfaction with social participation and social anchorage were associated with a high frequency of health care utilization |
| Roberts (2018)[71] | USA | High | 15 | Qualitative | Older adults | Care institution | NA | Grounded theory | Low | 1. The degree and reciprocity of disclosure and amount of engagement in shared times influenced strength and closeness of friendship. 2. Physical proximity could both positively and negatively influence the development of friendly peer relationships (common social space and private personal space). 3. Residents with dementia and communication difficulties were often not sought out for relationships or were avoided (difficulty of connecting with them). 4. There was a professional boundary between residents and health professionals |
| Rocha et al. (2009)[72] | Brazil | Upper-  Middle | 7 | Qualitative | Older adults | Community | NA | Thematic analysis | Low | 1. Children, friends, neighbours, religion as sources of support. 2. Emotional support came from children, wife and caregiver. This type of support is linked to care and attention they received from the closed ones. Information support came from health professionals. |
| Roe et al. (2001)[73] | USA | High | 20 | Qualitative | Older adults | Community | 79 | Thematic analysis | Moderate | 1. Elders' underlying medical conditions and related care influenced the help they needed with their instrumental activities of daily living and personal activities of daily living. 2. The majority of seniors needed and received help with shopping, preparing meals, housework, and travel away from home (e.g. daughters and daughters in law). Help with routine shopping was provided mainly by family (nine seniors). Staff would clean apartments once a week and change bed linen for seniors in assisted living or the nursing home. Half the seniors were able to take their own medication. Help with finances was often provided by children, a friend or spouse. A few residents received payment from social security or pension. |
| Rowe et al. (2006)[74] | USA | High | 522 | Quantitative | Older adults | Community | NA | Logistic regression | Low | 1. Social network size and instrumental support were not significantly associated with suicidal ideation. 2. “Satisfaction with relationships” (adjusted odds ratio: 0.59, 95% confidence interval: 0.39–0.90) and “feeling useful” (adjusted odds ratio: 0.50, 95% confidence interval: 0.35– 0.71) remained significantly associated with suicidal ideation in the final multivariate logistic regression model controlling for age, sex, educational status, race, living arrangement, marital status, cognitive status, depression, medical morbidity, and disability. |
| Saito et al. (2018)[75] | Japan | High | 13984 | Quantitative | Older adults | Community | 72.6 | Cox proportional hazards model | Moderate | 1. Respondents who had contact with friends and/or were married, participating in community groups, engaging in paid work and exchanging support with their family members were 11%–17% less likely to develop incident dementia. |
| Schenk et al. (2014)[76] | Netherlands | High | 3043 | Quantitative | Older adults | Community | NA | Multilevel modelling | Moderate | 1. Partnered adults had a ten times lower odds of receiving unskilled public care compared to those who were unpartnered. 2. Partnered women with poor health had higher odds of receiving public care than partnered men with poor health. 3. With every unit increase in the log of household income, the odds of receiving public care were 3.7 times smaller. 4. The odds of receiving unskilled public care did not differ by age of the respondent. 5. The odds of receiving some form of public care were 1.2 times larger with every year that men and women were older |
| Schmidt et al. (2021)[77] | 27 European countries and Israel | High | 102126 | Quantitative | Older adults | Community | 67.2 | Multilevel analysis | Low | 1. Most participants lived in rural areas or villages (34.3% and 32.8%) and in a free-standing one- or two-family house (42.3% and 44.6%). 2. People living in small towns with larger social network size had a significantly higher QoL than their rural counterparts, while this difference was not significant for people living in small towns but with smaller social network sizes. |
| Schnettler et al. (2015)[78] | Germany | High | 1886 | Quantitative | Older adults | Community | NA | Descriptive and regression analyses | Moderate | 1. The childless (3.38) have smaller networks than remote (4.51) and regional parents (4.47). 2. Remote parents have fewer children and grandchildren in their personal networks, but more friends and extended kin, than regional parents who have at least one child close by. Thus, the overall network size for both types of parents is about equal. 3. For the childless, the increases in personal network size and the number of potential supporters seem mainly driven by an increase in the number of friends and extended family members. For parents, we find that children (and grandchildren) play a decreasing role as potential supporters across cohorts. This decrease is balanced by an increasing importance of extended kin, friends and acquaintances, thus leaving the size of support networks relatively stable across cohorts. 4. For the childless, the effect each extended family member has on the number of available informational supporters is significantly stronger than it is for regional parents. But there is no statistically significant difference in comparison to remote parents.5. Taken together, these results indicate that the childless partially substitute for the non-existing social support of children through a higher tie efficiency of their extended kin and friendship ties. The effects of extended kin are similar for the childless and remote parents across all cohorts |
| Siette et al. (2020)[79] | Australasia | High | 175 | Quantitative | Older adults | Community | 80 | Multiple regression | Moderate | 1. Participants had a mean social network score of 34 (out of a possible 60) and thus were not socially isolated. 2. The type, frequency and number of social interactions with friends or family were not statistically different for participants with or without cognitive impairment. 3. Respondents aged 65-69 years had little problems with self-care (6.3%) but this increased to 16.7% in respondents aged between 75 to 79 years old. In older adult aged 85 years or more, mobility was the most frequently reported problem (45.6%), while self-care was least frequently reported as a problem (10.3%). The proportion of persons reporting problems, and the number of domains with problems rose with increasing age. In all age-groups and for all five domains, individuals with cognitive impairment reported more problems than individuals without cognitive impairment. 4. EQ-5D-5L index values were strongly associated with cognitive status score (higher score; no cognitive impairment). 5. The final model showed that men (ß = -2.84, p = 0.01) with higher levels of education (ß = 0.84, p = 0.03) and with high social networks (ß = 0.18, p = 0.04), receiving fewer service hours (ß = -0.14, p<0.001), and more service types (ß = 1.8, p = 0.02) had higher cognitive scores |
| Sintonen et al. (2014)[80] | Finland | High | 1680 | Quantitative | Older adults | Community | NA | Structural path analysis | Moderate | 1. Social disability (difficulty creating and maintaining relationships) was negatively related to the stability of social relationships. 2. The distant social network (not close to friends) was negatively related to loneliness together with social disability. 3. Social disability and loneliness increase the need for care. 4. The indirect effects of social networks (intensity) through social stability, disability and loneliness on acute care needs were statistically significant. Stronger social networks could decrease the need for acute care after sudden life changes for elderly individuals. |
| Spillman et al. (2020)[81] | USA | High | 1288 | Quantitative | Older adults | Community | NA | Multinomial regression | Moderate | 1. Older adults with dementia more often had multiple caregivers than those without dementia (77.8% vs 71.7%). 2. The difference in caregiver task sharing for older adults with and without dementia was most pronounced for medical assistance (32.8% vs 16.4%) and mobility or self-care assistance (39.4% vs 22.5%). |
| Stacey-Konnert et al. (1992)[82] | USA | High | 50 | Mixed methods | Older adults | Community | 80.9 | Correlation test | Moderate | 1. Residents reported social networks of 8.2 members, comprising kin (2.46), non-kin residing in the CCRC (3.94), and non-kin residing elsewhere (1.76). 2. Family members were the preferred source of confidant relationships. 3. Age was not related to the size of non-kin networks in the CCRC. 4. Older residents reported fewer social ties to non-kin living elsewhere. 5. None of the respondents saw his or her children on a daily basis, 30% saw them once a week, and one third of the respondents saw their children less than once a month. Telephone contact was made primarily on a daily (17%) and weekly (66%) basis. 6. Spouses were the family members most often identified as giving and/or receiving assistance. 7. These confidant relationships tended to be reciprocal in nature. 8. 94% were identified non-kin in their social networks. 9. No differences in the size of non-kin non-community networks between those with friends living afar and close by. 10. The correlation between age and the size of the non-kin community network was not significant |
| Stafford et al. (2018)[83] | UK | High | 2132 | Quantitative | Older adults | Community | NA | Poisson regression | Moderate | 1. Small networks (especially less than three friends and relatives), not married and not having children were associated with increased non-participation in health services. 2. Frequent contact and high social relationship quality with the closest person were associated with low relative risk of non-participation in health services 3. We found no evidence that associations between social connectedness at ages 68–69 years and participation in preventive health services differed between men and women |
| Sta. Maria et al. (2017)[84] | Philippines | Lower-Middle | 6 | Qualitative | Older adults | Community | NA | Thematic analysis | High | 1. The themes of support include instrumental support, emotional care, social connectedness, and companionship during engagement in activities. Themes characterising lack of support include disrespect and lack of understanding, constraining one’s actions, helplessness in responding to the other’s needs, non-dependability and non-reliability, difficulty in maintaining social connections, making it difficult to play a desired or expected role. 2. Acquaintances provide the older person with an ongoing, long-time friendship. Often times these acquaintances would be old friends or members of the same community (church) |
| Palo Stoller et al. (1991)[85] | USA | High | 79 | Quantitative | Older adults | Community | 78.1 | Structural path analysis | Moderate | 1. Older people who reported higher levels of functional impairment received help with a broader scope of tasks. 2. Neither health status nor scope of assistance influences network size. This finding suggests that network size is unstable over time. 3. Older people's preference for a spouse as a caregiver, especially as needs for help escalate. 4. Respondents who were married, had smaller networks, and received a broader range of assistance re- ported fewer unmet needs |
| Suanet et al. (2019)[86] | Netherlands | High | 2151 | Quantitative | Older adults | Community | 80 | Logistic regression | Moderate | 1. Those in the restricted network and the family-focused network without a partner still received informal care less often than those in the family-focused network with a partner. 2. Those in wider community-focused diverse networks were now equally likely to use informal care, which can be explained by their lower functional limitations and better cognitive functioning. 3. Those in wider community-focused diverse networks also have better functional capacity and cognitive functioning. 4. Those in the family-focused network without a partner and the restricted network used formal care most often. 5. Those in a family-focused network with a partner were least likely to use formal care. 7. Individuals in a restricted network were most likely to use privately paid help. |
| Sullivan et al. (2021)[87] | USA | High | 13 | Qualitative | Older adults | Community | 70.2 | Thematic analysis | Low | 1. Respondents who discussed having social support and connections to family, friends, and/or the community following the transition from nursing home to home/community most commonly reported a positive sense of health and well-being compared to those who reported poor social connections. 2. They were often able to connect with their family via phone. 3. Functional decline was a potential barrier to access to social connections. Decreased mobility following discharge contributed to physical isolation and restrictions for some, which created challenges for maintaining psychosocial well-being. 4. in-person healthcare appointments, home-based healthcare, and other types of healthcare contacts were generally seen as beneficial in terms of social connection and social support. 5. External stressors affected quality of social connections (e.g. conflicts and financial burden) |
| Tang et al. (2011)[88] | USA | High | 4611 | Quantitative | Older adults | Community | NA | Ordinary least squares (OLS) regression | Moderate | 1. Living more than two hours away from child (OR = 0.77, p < .01) was associated with lower expectations of receiving regular help in comparison with people having no child. 2. Knowledge of home- and community-based services (HCBS) availability was associated with respondents reporting an older age at which they expected regular help (services) and moving. 3. Information source of family/friends was important for aging in place expectation. 4. living with child was associated with lower odds of never moving compared with individuals without child |
| Teerawichitchainan et al. (2015)[89] | Vietnam, Thailand, Myanmar | Lower-Middle | 21367 | Quantitative | Older adults | Community | 70.1 | Ordinary least squares (OLS) regression | Moderate | 1. The impact of living with children on older adults' well-being did not statistically significantly different from that of other network arrangement in Vietnam and Thailand. However, a significant difference was found in Myanmar. 2. The significance of financial transfers from children to older parents was found to improve their psychological wellbeing. 3. Physical visits from non-co-resident children were significantly associated with improved mental wellbeing among elderly parents across all settings. |
| Tolkacheva et al. (2011)[90] | Netherlands | High | 602 | Quantitative | Older adults | Community | 79.6 | Structural path model | Moderate | 1. An adult child experienced lower care-giver burden when the informal care-giving network size was larger, when more types of tasks were shared across the network. 2. One-third of care recipients had (early stage) dementia, and 9 per cent had psychiatric problems; 14 per cent of parents could not be left alone longer than half-an-hour. The estimates of parental physical limitations were relatively high. 3. The average size of care-giving network was 2.8 and ranged from one to nine. 4. Most of the tasks that an adult child carried out were shared with at least one other care-giver (e.g. partner, own child, non-kin caregiver, and siblings). 5. More cognitive and physical impairments, as well as more hours of informal care, were positively correlated with an adult child’s care-giver burden. 6. the longer that others shared, in the responsibility for care, the lower an adult child’s care-giving burden |
| Verbeke (2021)[91] | Belgium | High | 1556 | Quantitative | Older adults | Community | NA | Descriptive analysis | High | 1. The large majority of hospital deaths (73%) had family members among their witnesses, only slightly less than elderly who died in private houses (81%). Almost half (45% to 46%) of family witnesses were children (-in-law), though cousins were also relatively important (20% and 23%). 3. Family relations also extended beyond the nuclear households. Cousins, siblings, nieces or nephews each made up between 10% and 14% of family witnesses. 4. Nonfamily witnesses, specifically neighbours and friends, also appeared to play a role, especially for specific groups such as single women. Distant neighbours and friends together made up at least 16% to 27% of non-familial witnesses Proximity appeared to be a crucial factor fostering amicable informal relations between friends. |
| Vos et al. (2019)[92] | Netherlands | High | 34 | Qualitative | Older adults | Community | NA | Thematic analysis | Low | 1. Women continued to spend time with friends after the loss of their partner, whereas men tended to spend more time with their (grand) children. 2. Maintaining existing friendships appeared to be challenging, because of the decreased mobility and dependency on carers. 3. Distance limits older adults in maintaining contact with those they have known for so long. A location nearby to meet others and the help of someone who connected people and helped older adults to overcome their barriers 4. Older adults should not rely on their children was a shared belief, because the children have their own lives. 5. In high‐SES or city families, older adults and their children frequently relied on professionals for support, whereas low‐SES or rural families seem to have more people in their neighbourhood who support them. 6. When families were living afar, older adults connected with them via email or WhatsApp. 7. Older adults try to reduce dependency by maintaining reciprocity. 8. Life could be tough for older adults when experiencing social network change, but that they tended to find ways to recover to a certain extent. |
| Wenger (1993)[93] | UK | High | 30 | Qualitative | Older adults | Community | 79 | Thematic analysis | Moderate | 1. Most elderly persons resisted seeking or accepting help with daily chores if they could possibly do them themselves. 2. Family help took place in the context of reciprocity. 3. Elderly parents were careful to take account of other responsibilities and pressures in their children’s lives. 4. They tend to do more for siblings who are childless or ill and expect less of those in poor health. 5. Grandchildren, if involved at all, typically help their own parents to care for the grandparents rather than provide direct support. 6. Relationships with nieces, nephews and cousins are mainly symbolic and limited to occasional visits and maintenance of contact. 7. While numbers and types of family relationships are limited by family size and ties of blood or marriage, numbers of friends and involvement with neighbours are much more a matter of choice. 8. Most of the elderly people interviewed were involved in the community in a number of ways: more than four-fifths (82%) claimed membership of a religious group and almost half (49%) belonged to voluntary organizations (other than religious groups). 9. Telephone contact with distant family members is an important reciprocal source of emotional support and advice. 10. Those with wider community focused networks were the most likely to feel that emergency help would be forthcoming from the community but the least likely to think that regular help would be available. |
| Wiersma (2008)[94] | Canada | High | 34 | Qualitative | Older adults | Care institution | NA | Thematic analysis | Moderate | 1. Embedded within this restrictive context are two types of relationships—functional relationships and supportive relationships. Relationships with staff who attended to people’s medical and physical needs appeared to be functional and task-oriented. 2. Not only did participants describe a close relationship with some staff, but such relationships appeared to be reciprocal. |
| Wiles et al. (2019)[95] | New Zealand | High | 76 | Qualitative | Older adults | Community | NA | Thematic analysis | Low | 1. The befriending service helped alleviate older adults' social isolation and loneliness and that supportive services to foster connection are needed. 2. The sense of mutual exchange was an important aspect of a genuine, meaningful relationship for both service users and volunteers. 3. Service-users and volunteer visitors also stressed reliability in relation to the confidential nature of conversation and the ability of visitors to support people with a variety of serious issues, including agoraphobia, depression, and suicidal feelings |
| Williams et al. (2002)[96] | USA | High | 187 | Quantitative | Older adults | Community | 74 | Hierarchical linear regression | Moderate | 1. As network size increased, caregivers perceived that they had more informal social support. 2. Caregivers were more likely (OR 5 1.17, 95% CI 5 1.03–1.34, p 5 .017) to use formal support when they cared for care recipients with higher levels of IADL limitations. |
| Wu et al. (2019)[97] | China | Upper-  Middle | 485 | Quantitative | Older adults | Community | 70.1 | Path analysis | Moderate | 1. Neighbours network (β=0.22, p < 0.05), friend network (β=0.13, p < 0.05) and family network (β=0.20, p < 0.05) positively affected healthy aging (e.g. self-care), health-promoting behaviour and self-efficacy. |
| Xu et al. (2019)[98] | China | Upper-  Middle | 786 | Quantitative | Older adults | Community | 74.9 | Hierarchical linear regression | Low | 1. All three groups of older adults, being emotionally close with children was correlated with lower levels of worry. 2. The benefits of having some local friends in reducing worry were stronger among older adults in immigrant families than those in non-migrant families |
| Yoo et al.(2012)[99] | USA | High | 14 | Qualitative | Older adults | Community | NA | Thematic analysis | Low | 1. All of the Korean elderly immigrants in this sample reported that their close relationships were Korean-based. 2. Eight respondents reported that neighbours, in particular Korean neighbours, were one of their primary sources of emotional and instrumental support. 3. These relationships with Korean neighbours evolved informally as they sought others who shared their language and cultural traditions. 4. The respondents mentioned that they received comfort from the day-care because they could get out of their housing and go somewhere every day. 5. There was an overwhelming expression of support for the quality of services, and the precious chance to get out of their housing and meet others. 6. The majority of respondents (n=13) reported that they had a close relationship with their adult children but lived independently from them. 7. Their main financial source was social assistance, and that they did not expect financial assistance from their children. 8. They did not want to go to great lengths to reach out to their children for support, because their children did not live in the neighbourhood and have their professional lives. 8. Most said either that they lived with their spouse because they had no choice, or to maintain their legal marriage status. 9. Twelve had close connections with the Korean church and church members, giving instrumental support including social contacts and a sense of community |
| Zhang et al. (2018)[100] | China | Upper-  Middle | 205 | Quantitative | Older adults | Care institution | 77.3 | Descriptive and correlation tests | Moderate | 1. Participants with poorer physical health but higher social support (support from friends, family and significant others) reported less suicidal thoughts than those with poorer physical health and lower social support |
| Cheng et al. (2022)[101] | Singapore | High | 2738 | Quantitative | Older adults | Community | NA | Latent transition analysis | Low | Diverse social network profiles were observed. Compared to sustained diverse profiles over time, sustained restricted profiles or a transition from diverse to restricted profiles entailed increased mortality risk. The mortality risk following a restricted-to-diverse profile transition could be as low as that associated with sustained diverse profiles. |
| Cohen (2022)[102] | USA | High | 249 | Quantitative | Older adults | Community | 61.4 | Non-parametric analysis | Low | People with schizophrenia were less likely to have support from family than the comparison group, but have more nonkin and formal support. |
| Cohn-Schwartz et al. (2021)[103] | Austria, Germany, Sweden, Netherlands, Spain, Italy, France, Denmark, Switzerland, Belgium, Czech Republic, Slovenia and Estonia | High | 50071 | Quantitative | Older adults | Community | 66 | Latent class analysis | Moderate | Participants in family-poor network types had poorer cognition scores at follow-up, compared to those in the close family network, while those in multi-tie networks had consistently better scores. The family-rich network and the friend-enhanced network also had somewhat better cognitive function. |
| York Cornwell et al. (2021)[104] | USA | High | 3735 | Quantitative | Older adults | Community | NA | Logistic regression | Low | Kin ties are also more interconnected with the network  than are non-kin ties, regardless of geographic proximity. But within kin and non-kin ties, geographic proximity  matters. Older adults who reside in urban areas are significantly  more likely to include local friends and neighbors in  their networks, compared to those who live in small town  or rural areas. Compared to those in very affluent tracts,  older adults in high poverty tracts have nearly 90% higher  odds of naming a local friend or neighbor in their network. |
| Domènech‐Abella et al. (2021)[105] | Netherlands | High | 3107 | Quantitative | Older adults | Community | NA | Structural equation modelling | Moderate | Social network size (Coef. = −0.02; p < 0.05), predicted higher levels of loneliness, which predicted an increase in depressive symptoms (Coef. = 0.17; p < 0.05) |
| Guadalupe et al. (2022)[106] | Portugal | High | 612 | Quantitative | Older adults | Community | 75.6 | Clustering | Moderate | The most frequent are family networks (61.8%), constituted by 94.6% of family ties, on average, attesting the familistic nature of the older persons’ networks in Portugal, followed by friendship networks (23.5%) and neighbourhood networks (11.9%). The less frequent type is the institutional network (2.8%), dominated by formal ties (M = 59.3%). Sociographic profiles reveal that family networks are more likely to be held by middle-old focal subjects,  married or widowed, and with children. Friendship and neighbourhood networks are held by young-old subjects with different marital status, many of them living alone, with  a higher proportion of men with friendship networks. Institutional networks are held by old–old, widowed or single with no children. |
| Hamlin et al. (2022)[107] | USA | High | 465 | Quantitative | Older adults | Community | 63.59 | Analysis of variance | Low | There was a link between social activity participation  and episodic memory |
| Holcomb et al. (2022)[108] | USA | High | 37 | Qualitative | Older adults | Community | NA | Thematic analysis | Low | The diverse/no children network was composed of a large social network comprising family, friends, and many times a partner. The participants in this network type were more involved in the SGM community than those in other network types. |
| Kim et al. (2022)[109] | South Korea | High | 1724 | Quantitative | Older adults | Community | 72.91 | Hierarchical linear regression | Low | Older adults who do not live with spouses showed high levels of perceived loneliness |
| Litwin et al. (2022)[110] | Israel | High | 33485 | Quantitative | Older adults | Community | 65.1 | Multivariate logistic regression | Moderate | Face-to-face network contact significantly reduced negative mental health changes while electronic contact significantly increased them. There was no observed effect of electronic contact on change in depressed state. Electronic contact, on the other hand, had no association with post-outbreak depression and a slightly positive association with anxiety. |
| Lottmann et al. (2022)[111] | UK | High | 175 | Quantitative | LGBT older adults | Community | NA | Descriptive statistics | Moderate | A spouse/partner was viewed as significant person for all  categories of support, although it is very notable that friends were also viewed as the most relevant source for emotional support and practical help. Respondents were most comfortable (64%) with health professionals |
| McCausland et al. (2021)[112] | Ireland | High | 560 | Quantitative | Older people with disability | Community | NA | Descriptive statistics | Moderate | 92.4% (560/606) reported that they had friends. Co-resident  friends were the most reported (71.8%, 402/560). Those whose best friend was a carer/service provider and were satisfied with life had the highest predicted mean friendship quality score |
| Meister et al. (2022)[113] | USA | High | 2553 | Quantitative | Older adults | Community | 76.63 | Linear regression | Moderate | All correlations between social variables and cognition  variables, if significant, were small |
| Nie et al. (2021)[114] | Czech Republic, Poland, Russia | High | 6691 | Quantitative | Older adults | Community | 62.2 | Linear regression | Low | The study showed positive associations of global cognitive  function with social activity participation and network size of friends and relatives, but not with contact frequency  in either network. |
| Park et al. (2021)[115] | South Korea and USA | High | 1495 | Quantitative | Older adults | Community | NA | Multiple regression | Moderate | Neither family network nor friend network was associated with depressive symptoms but living alone and low perceived financial  status was. |
| Parkhurst et al. (2022)[116] | USA | High | 425 | Quantitative | Older adults | Community | 70.82 | Latent class analysis | Moderate | The “moderate-sized, friend-oriented network” class reported  having an average of 3 confidants, primarily composed of  non-relatives, and most often reported living alone, having  weekly contact with confidants, relatively low closeness  with them, and the lowest likelihood of discussing health  with them. The friend-oriented class reported the greatest loneliness, perceived burden, and lifetime prevalence of suicidal ideation and attempts than members of the “average-sized, close network” class |
| Rhee et al. (2021)[117] | USA | High | 5799 | Quantitative | Older adults | Community | 73.8 | Multinomial regression | Low | Older adults with low diversity of social networks were older, and had lower income and education levels when compared to those with high diversity of social networks. Older adults with high diversity of social networks (i.e., Groups 2 and 4) had higher mean scores of both mental component summary (MCS) and physical component summary (PCS) of HRQOL and quality-adjusted life years (QALYs) when compared to those with low diversity of social networks. |
| Saito et al. (2021)[118] | Japan | High | 808 | Quantitative | Older adults | Community | 72 | Regression | Moderate | Social network diversity was significantly associated with MMSE score decline. |
| Stephens et al. (2022)[119] | New Zealand | High | 917 | Quantitative | Older adults | Community | 75 | Hierarchical multiple regression | Moderate | Neighborhood variables added significant explanation of variance in both social and emotional loneliness. The effects of all neighborhood variables were mediated by Private-Restricted on Social Loneliness only |
| Štípková (2021)[120] | Czech Republic | High | 3934 | Quantitative | Older adults | Community | NA | Regression | Moderate | Size of the network ranges from 0 to 7. The average size is 2.5 persons. A health condition that limits one’s usual activities increases the loneliness score. Having children reduces loneliness by 7.3 percentage points. |
| Sung et al. (2022)[121] | Singapore | High | 1305 | Quantitative | Older adults | Community | 70.63 | Multinomial logistic regression | Low | Older adults in the restricted type were unmarried,  more than half of them lived alone, and two-thirds had  no children. They were less likely to interact with relatives  and friends or have community participation. Older adults in the restricted type were more likely to live in a small house and have a greater extent of depressive symptoms |
| Torres et al. (2022)[122] | Northern Europe (Denmark and Sweden), western Europe  (Austria, Germany, France, the Netherlands, Switzerland,  Belgium, Ireland, and Luxembourg), southern Europe (Spain,  Italy, Greece, and Portugal), and eastern Europe (Czech  Republic, Poland, Hungary, Slovenia, Estonia, and Croatia) | High | 27272 | Quantitative | Older adults | Community | 74.95 | Analysis of  Variance | Moderate | The “no network” group presents worse health outcomes in all variables. The “friends” network is more protective toward cognitive functioning and physical health and the “spouse” and “family” ones are more protective toward mental health. |
| Xin et al. (2022)[123] | China | Upper_Middle | 8669 | Quantitative | Older adults | Community | 69.51 | Structural equation modelling | Moderate | Health risks had significant effects on the psychological and social mechanisms of older adults’ social participation. |
| Yoo-Jeong et al. (2022)[124] | USA | High | 146 | Quantitative | Older adults with HIV | Community | 56.53 | Multivariate regression | Moderate | Regular contact occurred most frequently with friends (82%)  and relatives (77%). Participants with limited social networks (SNI < 4) were more likely to have higher scores for depressive symptoms, HIV-related stigma, and loneliness, and reduced emotional well-being than individuals with diverse social network  (p < .05). |
| Zhang et al. (2022)[125] | China | High | 1363 | Quantitative | Older adults | Community | 69.29 | Ordinary least squares (OLS)  regression | Moderate | The results show that Internet use positively and significantly impacts the social networks of the elderly. Compared to the elderly who do not use the Internet, the elderly who use the Internet have a larger social network size, more significant social network heterogeneity |

# Table S4: Definition of network-related concepts

| **Node** | **Category** | **Definition** |
| --- | --- | --- |
| Intensity of networks | Networks | Strength or closeness of relationship with people and the society |
| Social/Community engagement | Networks | Participation in formal community services (e.g. organised by the third sector) or informal social activities (e.g. attending informal social events) |
| Size of networks | Networks | The number of support resources an older adult has |
| Diversity of networks | Networks | Variety of relationships in the network |
| Reciprocity/Mutuality | Networks | Mutual interest, dependence, influence or actions (e.g. sharing) |
| Frequency of contacts | Networks | Contact frequency (physical or non-physical) between older adults and their support networks |
| Duration of relationship | Networks | The length of relationship |
| Informal care support | Informal | Unpaid care or support provided by family members, friends, or neighbours |
| Geographical/Physical proximity | Space | Geographical distance to older adults’ support networks |
| Social space | Space | Physical or virtual space where people gather and interact |
| Communication technology | Medium | Technology used to communicate information |
| Government policy | Context | Laws, regulations, actions and decisions made by public authorities to promote wellness of people (e.g. university credit, national health insurance, or pension) |
| Stigma | Context | Attribute that is discrediting in the society |
| Age | Characteristics | The length of time that older adults have lived |
| Socio-economic status | Characteristics | A social standing of an individual based on social class and income |
| Heath status | State | Medical conditions |
| ADL limitations/Physical disability | State | Mobility problems or difficulty of carrying out activities of daily living |
| Mental wellbeing | State | A state of mental wellbeing |
| Cognitive impairment | State | A condition in which an older adult has problems with cognition |
| Care burden | State | Distress experienced by a carer as a result of caring for an older adult |
| Quality of life | State | Subjective measure of an older adult’s perception of different aspects of their life |
| Life satisfaction | State | Older adults’ favourable attitude towards their life |
| Health behaviour | Behaviour | Actions an older adult takes that affect their health |
| Healthcare use | Behaviour | Use of health services |
| Care decision making | Behaviour | Decision making on care for older adults |
| Formal care support | Formal | Support by a social and health care professional, trained carer, government, or institution |

# Table S5: Centrality score of network-related concepts in descending order

| Node | Category | Definition | Eigenvector centrality | Degree centrality |
| --- | --- | --- | --- | --- |
| Social/Community engagement | Networks | Participation in formal community services (e.g. organised by the third sector) or informal social activities (e.g. attending informal social events) | 1 | 21 |
| Intensity of networks | Networks | Strength or closeness of relationship with people and the society | 0.98 | 20 |
| Health status | State | Medical conditions | 0.76 | 17 |
| Formal care support | Formal | Support by a social and health care professional, trained carer, government, or institution | 0.76 | 19 |
| Mental wellbeing | State | A state of mental wellbeing | 0.63 | 11 |
| Geographical/Physical proximity | Space | Geographical distance to older adults’ support networks | 0.62 | 11 |
| Informal care support | Informal | Unpaid care or support provided by family members, friends, or neighbours | 0.6 | 16 |
| Diversity of networks | Networks | Variety of relationships in the network | 0.59 | 14 |
| Size of networks | Networks | The number of support resources an older adult has | 0.52 | 15 |
| Healthcare use | Behaviour | Use of health services | 0.52 | 16 |
| ADL limitations/Physical disability | State | Mobility problems or difficulty of carrying out activities of daily living | 0.51 | 9 |
| Frequency of contacts | Networks | Contact frequency (physical or non-physical) between older adults and their support networks | 0.39 | 9 |
| Reciprocity/Mutuality | Networks | Mutual interest, dependence, influence or actions (e.g. sharing) | 0.35 | 6 |
| Social space | Space | A recreational space where people can gather and interact | 0.32 | 3 |
| Age | Characteristics | The length of time that older adults have lived | 0.27 | 7 |
| Cognitive impairment | State | A condition in which an older adult has problems with cognition | 0.26 | 10 |
| Communication technology | Medium | Technology used to communicate information | 0.2 | 7 |
| Care burden | State | Distress experienced by a carer as a result of caring for an older adult | 0.19 | 7 |
| Socio-economic status | Characteristics | A social standing of an individual based on social class and income | 0.18 | 5 |
| Health behaviour | Behaviour | Actions an older adult takes that affect their health | 0.13 | 6 |
| Government policy | Context | Laws, regulations, actions and decisions made by public authorities to promote wellness of people (e.g. university credit, national health insurance, or pension) | 0.08 | 3 |
| Quality of life | State | Subjective measure of an older adult’s perception of different aspects of their life | 0.08 | 6 |
| Stigma | Context | Attribute that is discrediting in the society | 0.06 | 1 |
| Life satisfaction | State | Older adults’ favourable attitude towards their life | 0.06 | 3 |
| Care decision making | Behaviour | Decision making on care for older adults | 0.05 | 2 |
| Duration of relationship | Networks | The length of relationship | 0.03 | 2 |

# Table S6: Links between concepts identified by included studies

| **Links between** | | **Number of studies reporting the link** | **Findings** | **Study type** | **Normalised scale (0-1)** |
| --- | --- | --- | --- | --- | --- |
| **Concept01** | **Concept02** |  |  |  |  |
| Reciprocity/Mutuality | Intensity of networks | 13 | Reciprocity/mutuality was positively linked to the strength of relationship between older adults and their care networks | Qualitative studies[19, 53, 65, 66, 71, 84, 94, 95, 99]  Quantitative studies[33, 69]  Mixed methods[28, 82] | 1.0 |
| Geographical/Physical proximity | Informal care support | 12 | The shorter the distance between older adults and their networks, the higher likelihood of receiving informal care | Qualitative studies[31, 46, 52, 92, 99]  Quantitative design[35, 43, 57, 78, 88, 109, 126] | 0.9 |
| Social/Community engagement | Network diversity | 11 | Active social engagement was linked to increased network diversity | Qualitative studies[47, 84, 87, 93]  Quantitative studies[7, 29, 30, 62, 86, 88, 101] | 0.8 |
| Social space | Social/Community engagement | 9 | Creation of social space and social opportunity was positively linked to older adults’ social engagement | Qualitative studies[9, 12, 20, 32, 55, 59, 68, 71]  Mixed methods[40] | 0.7 |
| Social/Community engagement | Mental wellbeing | 9 | Engaging in community activities was positively linked to older adults’ mental wellbeing and a sense of connectedness with the society | Qualitative studies[16, 32, 68, 87, 95]  Quantitative studies[38, 80, 120]  Mixed methods[40] | 0.7 |
| Frequency of contact | Intensity of networks | 8 | An increase in frequency of contact was linked to deepening relationships and emotional closeness. On the other hand, limited physical and telephone contacts were reported as a barrier to building rapport and receiving support | Qualitative studies[31, 34, 35, 56]  Quantitative studies[33, 49, 50]  Mixed methods[82] | 0.6 |
| Age | Formal care support | 8 | Older age was associated with the increased use of or need for formal care support | Qualitative studies[35]  Quantitative studies[15, 50, 51, 54, 60, 63, 106] | 0.6 |
| Geographical/Physical proximity | Intensity of networks | 8 | The geographical distance between older adults and their care networks was negatively associated with older adults’ social ties. | Qualitative studies[9, 31, 32, 92]  Quantitative studies[10, 91, 116, 126] | 0.6 |
| Health status | Formal care support | 7 | Poor health status increased the likelihood of receiving formal care services | Qualitative studies[35, 59]  Quantitative studies[13, 36, 43, 60, 63] | 0.5 |
| Formal care support | Intensity of networks | 7 | Engaging in formal care services (health and social care services) was positively linked to social connectedness | Qualitative studies[12, 13, 19, 20, 87, 95, 99]  Quantitative studies[13] | 0.5 |
| ADL limitations/ Physical disability | Social/Community engagement | 7 | ADL limitations were negatively associated with social engagement | Qualitative studies[9, 32, 59, 87, 92]  Quantitative studies[38, 120] | 0.5 |
| Size of networks | Mental wellbeing | 7 | Having more support was positively associated with mental wellbeing | Quantitative studies[21, 45, 49, 74, 105, 122, 124] | 0.5 |
| Social/Community engagement | Intensity of networks | 6 | Older adults’ active social engagement with others (amount of engagement) was positively linked to the strength of relationships | Qualitative studies[9, 12, 32, 66, 71, 99]  Quantitative studies[80] | 0.4 |
| Socio-economic status | Government policy | 6 | Older adults with low socio-economic status were more likely to receive support from government policies (e.g. government financial support, universal credit, public paid care, social security and pension) | Qualitative studies[34, 46, 73, 99]  Quantitative studies[43, 48] | 0.4 |
| ADL limitations/Physical disability | Formal care support | 6 | ADL limitations were associated with the increased use of formal care services | Quantitative studies[13, 33, 35, 51, 54, 60] | 0.4 |
| Social/Community engagement | Formal care support | 6 | Engaging in community services was associated with an increased access to formal care services (e.g. referrals) | Qualitative studies[52, 55, 59]  Quantitative studies[29, 70] | 0.4 |
|  |  |  | Social participation was negatively associated with health care utilisation | Quantitative study[60] |  |
| Social/Community engagement | Health status | 6 | Engagement in community activities was positively associated with mental health (including depression) and healthy ageing | Qualitative studies[16, 68]  Quantitative studies[7, 38, 39, 103] | 0.4 |
| Intensity of networks | Mental wellbeing | 6 | Distant relationship with their care networks was associated with poor mental wellbeing, loneliness, or suicidal thoughts | Quantitative studies [23, 49, 80, 100, 116] | 0.4 |
| Informal care support | Mental wellbeing | 6 | Having informal care support was positively associated with mental wellbeing | Qualitative study[87]  Quantitative studies[21, 23, 89, 109, 120] | 0.4 |
| Intensity of networks | Health status | 5 | Being emotionally close to their children was associated with lower levels of worry | Quantitative study[98] | 0.3 |
|  |  |  | Close relationships with care networks were associated with better health status | Qualitative studies[87]  Quantitative studies[36, 100] |  |
|  |  |  | Emotional closeness was associated with alleviated depressive symptoms | Quantitative study^[49]^ |  |
| Social space | Intensity of networks | 5 | Creation of social space was associated with increased physical proximity and enhanced connectedness | Qualitative studies[9, 20, 65, 71]  Quantitative study[11] | 0.3 |
| ADL limitations/Physical disability | Healthcare use | 5 | As ADL limitations increased, the probability of using formal care services (paid services or institutionalization) increased | Quantitative studies[35, 48, 51, 54, 60] | 0.3 |
| Size of networks | Healthcare use | 5 | Older adults with larger networks were less likely to use healthcare services (including nursing home) | Quantitative studies[36, 60, 86] | 0.3 |
|  |  |  | Small network size was associated with low participation in health services | Quantitative study[83] |  |
|  |  |  | Older adults with limited access to family support had a higher chance of using emergency care | Quantitative study[22] |  |
| Social/Community engagement | Healthcare use | 5 | Older adults with a lack of participation in social activities were more likely to transition out of home and into an institution | Quantitative study[60] | 0.3 |
|  |  |  | Older adults using voluntary social care services had higher chance of being connected with specialized health care providers | Qualitative study[55] |  |
|  |  |  | High satisfaction with social participation was associated with high frequency of healthcare use | Quantitative study[70] |  |
|  |  |  | Social disability was associated with an increased use of health care | Quantitative study[80] |  |
|  |  |  | In-person healthcare appointments and home-based healthcare were reported to help older adults connect with the community | Qualitative study[87] |  |
| Diversity of networks | Care burden | 5 | Care tasks distributed to more than one carer was associated with reduced care burden | Qualitative study[24, 47, 73]  Quantitative study[44] | 0.3 |
|  |  |  | Long hours of informal care was positively associated with increased care burden | Quantitative study[90] |  |
| ADL limitations/Physical disability | Informal care support | 5 | Older adults with ADL limitations were more likely to receive informal care support | Quantitative studies[11, 18, 33, 57] | 0.3 |
|  |  |  | Physical decline was a barrier to social support for older adults | Qualitative study[34] |  |
| Size of networks | Informal care support | 5 | A larger network size was positively associated with the provision of informal care support | Qualitative study[28]  Quantitative studies[35, 50, 58, 96]  Mixed methods[34] | 0.3 |
| Communication technology | Frequency of contact | 4 | Older adults maintained frequent contacts with their family through telephone/mobile phone | Qualitative studies[31, 32, 56, 87] | 0.3 |
| ADL limitations/Physical disability | Intensity of networks | 4 | Functional decline was associated with decreased social connections and support (e.g. difficulty of maintaining relationships or emotional closeness) | Qualitative studies[34, 87, 92]  Quantitative study[18] | 0.3 |
| Size of networks | Intensity of networks | 4 | Network size was not significantly associated with building/deepening networks | Qualitative study[65]  Quantitative study[21] | 0.3 |
|  |  |  | As network size increased, older adults were more likely to have more support within the network | Quantitative studies[35, 96] |  |
| Informal care support | Health behaviour | 4 | Lack of support from family negatively influenced older adults’ active engagement in or motivation for the care or health promoting behaviour | Qualitative study[12]  Quantitative studies[21, 97] | 0.3 |
|  |  |  | Informal care support was associated with oral health behaviour | Quantitative study[5] |  |
| Age | Healthcare use | 4 | Old age was associated with an increased use of health services | Quantitative studies[15, 51, 54, 60] | 0.3 |
| Geographical/Physical proximity | Healthcare use | 4 | Older adults living in town were more likely to use healthcare services | Quantitative study[33] | 0.3 |
|  |  |  | Long distance between older adults and their care networks was associated with low utilization of health care | Qualitative study[31]  Quantitative studies[36, 88] |  |
| Health status | Healthcare use | 4 | Older adults with poor health status was less likely to return home from care settings | Quantitative study[13] | 0.3 |
|  |  |  | Poor health was significantly associated with frequent use of health care | Qualitative study[59]  Quantitative studies[36, 60] |  |
| Intensity of networks | Informal care support | 4 | Strength of relationship was positively associated with informal care support | Qualitative study[16, 84]  Quantitative studies[21, 57] | 0.3 |
| Size of networks | Formal care support | 4 | Increased network size was associated with the lower use of health care services | Quantitative studies[36, 86] | 0.3 |
|  |  |  | Small network size was associated with low participation in health services | Quantitative study[83] |  |
|  |  |  | Older adults with no social support (zero) had higher chance of being institutionalized | Quantitative study[60] |  |
| Socio-economic status | Formal care support | 4 | Older adults with high socio-economic status were more likely to receive care services provided by professionals or private care | Qualitative study[92]  Quantitative studies[42, 43, 48] | 0.3 |
| Informal care support | Health status | 4 | Older adults with poor relationships with family were more likely to have poor perceived health status | Quantitative study[23, 103] | 0.3 |
|  |  |  | Older adults receiving social support were more likely to have good health or psychological wellbeing | Qualitative study[87]  Quantitative study[89] |  |
| Geographical/Physical proximity | Frequency of contact | 4 | The geographical distance between older adults and their family negatively affected their frequency of contacts | Qualitative studies[31, 92]  Quantitative study[41, 126] | 0.3 |
| Health status | Social/Community engagement | 4 | Poor health status was negatively associated with social engagement | Qualitative study[9]  Quantitative study[120, 123]  Mixed methods[40] | 0.3 |
| Geographical/Physical proximity | Health status | 4 | Living alone was significantly associated with older adults’ wellbeing | Quantitative study[89, 115, 116] | 0.3 |
|  |  |  | Living with children was significantly associated with improved somatic symptoms | Quantitative study[6] |  |
| Social/Community engagement | Cognitive impairment | 4 | Social engagement was associated with lower prevalence of cognitive impairment and incident dementia | Quantitative studies[38, 75, 103, 107] | 0.3 |
| Cognitive impairment | Social/Community engagement | 3 | Cognitive impairment or dementia was negatively associated with social engagement | Qualitative studies[59, 71]  Quantitative study[38] | 0.2 |
| Diversity of networks | Intensity of networks | 3 | Older adults with diverse networks were more likely to have relationships that were intimate and meaningful | Qualitative study[9]  Quantitative studies[29, 30] | 0.2 |
| Communication technology | Intensity of networks | 3 | The use of communication technology (e.g. telephone and mobile apps) was reported to help maintain the strength of relationships | Qualitative studies[31, 34, 56] | 0.2 |
| Informal care support | Care decision making | 3 | Informal care support played an important role in decision making on older adult care | Qualitative study[24]  Quantitative study[48]  Mixed methods[28] | 0.2 |
| Socio-economic status | Healthcare use | 3 | Older adults with high socio-economic status were more likely to use private care or formal care services, whereas those with low socio-economic status were more likely to use publicly-funded care | Quantitative studies[43, 48, 76] | 0.2 |
| Intensity of networks | Healthcare use | 3 | Older adults with closer ties with their networks were less likely to use health and social care services | Quantitative studies[36, 37] | 0.2 |
|  |  |  | Network intensity was inversely related to returning home (from a care home) | Quantitative study[13] |  |
| ADL limitations/Physical disability | Care burden | 3 | More physical impairment was positively associated with care burden | Qualitative study[64]  Quantitative studies[85, 90] | 0.2 |
| Care burden | Formal care support | 3 | Increased care burden was positively associated with the use of formal care services | Qualitative studies[64, 73]  Quantitative study[44] | 0.2 |
| Geographical/Physical proximity | Formal care support | 3 | Older adults living in town were more likely to use formal care services | Quantitative study[33] | 0.2 |
|  |  |  | Formal care provision was unrelated to geographical distance (health and social care professionals were obliged to provide care regardless of the distance) | Qualitative study[31] |  |
|  |  |  | Long distance between older adults and their networks was associated with the low utilization of health care services | Quantitative study[36] |  |
| Formal care support | Mental wellbeing | 3 | Engaging in the animal companion programme had a positive effect on older adults’ mental wellbeing | Qualitative study[68] | 0.2 |
|  |  |  | Attending formal care services helped older adults to not feel lonely | Qualitative study[12] |  |
|  |  |  | Befriending services had a positive effect on older adults’ mental wellbeing | Qualitative study[95] |  |
| Geographical/Physical proximity | Communication technology | 3 | Older adults living far away from their family were more likely to use emails or mobile apps to maintain relationships with them | Qualitative studies[56, 92, 93] | 0.2 |
| Frequency of contact | Health status | 3 | Frequency of contact was significantly associated with improved somatic symptoms | Quantitative study[6] | 0.2 |
|  |  |  | Daily contact was negatively associated with the incidence of dementia | Quantitative study[26] |  |
|  |  |  | Face-to-face network contact significantly reduced negative mental health changes while electronic contact significantly increased them. | Quantitative study[110] |  |
| Stigma | Intensity of networks | 3 | Stigma worsened the strength of relationship between older adults and their networks | Qualitative studies[16, 59, 124] | 0.2 |
| Diversity of networks | Healthcare use | 3 | Having diverse networks was associated with less use of formal care services | Quantitative study[51] | 0.2 |
|  |  |  | Having a diverse network was more likely to be involved in community services | Qualitative study[108] |  |
|  |  |  | Having networks with similar traits was associated with less use of health care | Quantitative study[36] |  |
| Size of networks | Cognitive impairment | 3 | Large network size significantly reduced the incidence of developing dementia | Quantitative studies[26, 114] | 0.2 |
|  |  |  | There was no significant difference in network size between older adults with cognitive impairment and without | Quantitative study[79] |  |
| Diversity of networks | Formal care support | 3 | Having diverse networks was positively associated with the use of community services | Quantitative study[29]  Qualitative study[108] | 0.2 |
|  |  |  | Older adults with a diverse network were less likely to use formal care services (health care) | Quantitative study[51] |  |
| Size of networks | Health status | 3 | Older adults with a large network size were more likely to have better health status | Quantitative studies[36, 105, 122] | 0.2 |
| Diversity of networks | Social/Community engagement | 3 | Diversity of networks was positively linked with social engagement | Quantitative studies[29, 121]  Qualitative study[108] | 0.2 |
| Diversity of networks | Health status | 3 | Diverse networks were associated with reduced mortality risks or risks of developing dementia | Quantitative studies[101, 103, 118] | 0.2 |
| Geographical/Physical proximity | Mental wellbeing | 3 | Living with or close to family was less likely to develop mental health problems | Quantitative studies[109, 115, 116] | 0.2 |
| Diversity of networks | Mental wellbeing | 2 | Older adults with restricted networks were more likely to develop depressive symptoms | Quantitative studies[121, 124] | 0.1 |
| Socio-economic status | Social/Community engagement | 2 | Financial difficulties were negatively associated with social community | Qualitative study[87]  Quantitative study[17] | 0.1 |
| Frequency of contact | Social/Community engagement | 2 | Lack of contacts with the community was negatively associated with social engagement | Qualitative studies[34, 61] | 0.1 |
| Government policy | Healthcare use | 2 | Health policy (private or public funded care) was associated with older adults’ use of health care | Quantitative studies[43, 48] | 0.1 |
| Age | Size of networks | 2 | As older adults aged, the size of networks became smaller | Quantitative study[50]  Mixed methods[82] | 0.1 |
|  |  |  | Age was not associated with network size |  |  |
| Health status | Size of networks | 2 | Health decline was associated with a decrease in network size | Qualitative study[61]  Quantitative study[69] | 0.1 |
| Diversity of networks | Life satisfaction | 2 | Having diverse networks was positively associated with life satisfaction | Quantitative studies[14, 39] | 0.1 |
| Formal care support | Informal care support | 2 | Older adults receiving formal care support continued to rely on informal care support | Qualitative studies[31, 65] | 0.1 |
| Socio-economic status | Informal care support | 2 | Older adults with high socio-economic status were less likely to rely on spouse and children support | Qualitative study[92]  Quantitative study[42] | 0.1 |
| Age | Informal care support | 2 | The likelihood of receiving informal care support increased with age | Quantitative study[57] | 0.1 |
|  |  |  | As older adults aged, it was harder for them to make friends | Qualitative study[59] |  |
| Government policy | Formal care support | 2 | Health policy was associated with the availability and cost of formal care services | Qualitative study[52]  Quantitative study[48] | 0.1 |
| Communication technology | Diversity of networks | 2 | Internet access was associated with increased network diversity | Quantitative study[7, 125] | 0.1 |
| Geographical/physical proximity | Diversity of networks | 2 | Where older adults lived had an impact on whether they can meet and connect with people | Qualitative study[92]  Quantitative study[126] | 0.1 |
| Frequency of contact | Mental wellbeing | 2 | Frequency of contacts was positively associated with mental wellbeing | Quantitative study[49, 110] | 0.1 |
| Diversity of networks | Health behaviour | 1 | Increased diversity of networks was associated with oral health behaviour | Quantitative study[5] | 0.0 |
| Size of networks | Health behaviour | 1 | Network size was associated with oral health behaviour | Quantitative study[5] | 0.0 |
| Age | Health status | 1 | Old age was associated with poor health status | Quantitative study[6] | 0.0 |
| Communication technology | Mental wellbeing | 1 | Telephone contact with distant family provided emotional support | Qualitative study[93] | 0.0 |
| Health behaviour | Health status | 1 | Participation in physical activities was significantly associated with healthy ageing | Quantitative study[7] | 0.0 |
| Formal care support | Quality of life | 1 | Provision of formal care services at home to those in need was positively associated with quality of life | Quantitative study[7] | 0.0 |
| Informal care support | Quality of life | 1 | Provision of informal care support at home was positively associated with quality of life | Quantitative study[7] | 0.0 |
| Duration of relationship | Intensity of networks | 1 | Long duration of relationship was associated with its lasting quality | Qualitative study[9] | 0.0 |
| Health status | Intensity of networks | 1 | Poor health status negatively influenced social connection | Qualitative study[9] | 0.0 |
| Intensity of networks | Formal care support | 1 | Stronger social ties decreased the likelihood of receiving acute care | Quantitative study[80] | 0.0 |
| Intensity of networks | Health behaviour | 1 | Strength of relationship with care networks had an impact on older adults’ health behaviour | Qualitative study[16] | 0.0 |
| Formal care support | Health behaviour | 1 | Poor relationship with health professionals was negatively associated with older adults’ health behaviour | Qualitative study[16] | 0.0 |
| Reciprocity/mutuality | Informal care support | 1 | Reciprocity (sharing) was associated with the acquisition of social support | Qualitative study[53] | 0.0 |
| Frequency of contact | Cognitive impairment | 1 | Daily contact significantly reduced the risk of developing dementia | Quantitative study[26] | 0.0 |
| Size of networks | Reciprocity/mutuality | 1 | The more support older adults received, the more likely they had a shared role in decision making | Mixed method[28] | 0.0 |
| Frequency of contact | Healthcare use | 1 | Frequent contacts were associated with the less use of health care services | Quantitative study[36] | 0.0 |
| Duration of relationship | Healthcare use | 1 | Long duration of relationship was associated with the less use of health services | Quantitative study[36] | 0.0 |
| Social/Community engagement | ADL limitations/physical disability | 1 | Social engagement was significantly associated with lower prevalence of physical disability | Quantitative study[38] | 0.0 |
| Social/Community engagement | Quality of life | 1 | Social engagement was associated with better quality of life | Quantitative study[38] | 0.0 |
| Social/Community engagement | Life satisfaction | 1 | Non-integrated social network (family dependent and non-participation in community activities) was associated with a decreased odds of life satisfaction | Quantitative study[39] | 0.0 |
| Geographical/physical proximity | Social/Community engagement | 1 | Physical proximity was associated with an increased opportunity of helpful exchanges | Qualitative study[66] | 0.0 |
| Geographical/physical proximity | Care decision making | 1 | Informal caregivers living with older adults were more likely to be involved in care decision making | Quantitative study[44] | 0.0 |
| Communication technology | Informal care support | 1 | Limited telephone use created a barrier to informal care support | Qualitative study[34] | 0.0 |
| Reciprocity/mutuality | Social/Community engagement | 1 | Older adults’ perceived connectedness with people was associated with social engagement | Qualitative study[32] | 0.0 |
| Healthcare use | Informal care support | 1 | In-personal healthcare or home-based healthcare was seen by participants as beneficial for connections and support from family | Qualitative study[87] | 0.0 |
| Social space | Formal care support | 1 | Having community programmes in place helped with the increased use of formal care support | Qualitative study[55] | 0.0 |
| Age | Social/Community engagement | 1 | Old age was linked with a decrease in social engagement | Qualitative study[59] | 0.0 |
| Cognitive impairment | Formal care support | 1 | Older adults with cognitive impairment or probable dementia were more likely to be institutionalized | Quantitative study[60] | 0.0 |
| Cognitive impairment | Healthcare use | 1 | Older adults with cognitive impairment or probable dementia were more likely to be institutionalized | Quantitative study[60] | 0.0 |
| Health status | Frequency of contact | 1 | As older adults’ health declined, they had less social contacts | Qualitative study[61] | 0.0 |
| Size of networks | Social/Community engagement | 1 | Levels of social engagement depended on the number of ties older adults had outside the institution | Qualitative study[65] | 0.0 |
| Health status | ADL limitations/physical disability | 1 | Older adults’ underlying conditions were linked to their need for ADL | Qualitative study[73] | 0.0 |
| Intensity of networks | Cognitive impairment | 1 | Older adults with close ties with family and friends were less likely to develop dementia | Quantitative study[75] | 0.0 |
| Size of networks | Quality of life | 1 | People living in a small town with a larger network size was significantly associated with improved quality of life | Quantitative study[77] | 0.0 |
| Age | ADL limitations/physical disability | 1 | Old age was related to self-care problems | Quantitative study[79] | 0.0 |
| Cognitive impairment | Quality of life | 1 | Cognitive impairment was associated low quality of life | Quantitative study[79] | 0.0 |
| Mental wellbeing | Healthcare use | 1 | Loneliness was linked to the need for acute care | Quantitative study[80] | 0.0 |
| Health status | Care burden | 1 | Poor health status was associated with increased care burden | Quantitative study[90] | 0.0 |
| Care burden | Healthcare use | 1 | Increased care burden was associated with the frequent use of formal care | Quantitative study[44] | 0.0 |
| Size of networks | Care burden | 1 | An increase in care network size was linked with reduced care burden | Qualitative study[24] | 0.0 |
| Cognitive impairment | Care burden | 1 | Older adults with dementia were significantly more likely to have multiple caregivers than those without dementia | Quantitative study[81] | 0.0 |
| Informal care support | Reciprocity/mutuality | 1 | Informal care support showed greater instances of reciprocal relationships | Qualitative study[65] | 0.0 |
| Reciprocity/mutuality | Mental wellbeing | 1 | Perceived support was positively associated with mental wellbeing | Quantitative study[21] | 0.0 |
| Communication technology | Size of networks | 1 | Older adults who used internet had a large network size | Quantitative study[125] | 0.0 |
| Diversity of networks | Quality of life | 1 | Older adults with a diverse network were significantly more likely to have higher quality of life | Quantitative study[117] | 0.0 |
| Diversity of networks | Cognitive impairment | 1 | Older adults with a diverse network were less likely to develop cognitive impairment | Quantitative study[118] | 0.0 |
| Formal care support | Life satisfaction | 1 | Having support from health care providers were more likely to feel satisfied with life | Quantitative study[112] | 0.0 |

# Table S7: Lists of concepts within the three communities

| **Community group** | **Variable/Concept** |
| --- | --- |
| 1 | Communication technology |
|  | Mental wellbeing |
|  | Informal care support |
|  | Stigma |
|  | Network size |
|  | Reciprocity |
|  | Care decision making |
|  | Health behaviour |
|  | Intensity of networks |
|  | Frequency of contact |
|  | Proximity |
| 2 | Network diversity |
|  | ADL limitations |
|  | Social space |
|  | Life satisfaction |
|  | Cognitive impairment |
|  | Social engagement |
|  | Quality of life |
| 3 | Formal care support |
|  | SES |
|  | Policy |
|  | Health care use |
|  | Age |
|  | Health status |
|  | Care burden |
|  | Duration of relationship |

# Figure S1: Risk of bias assessment for qualitative studies

**
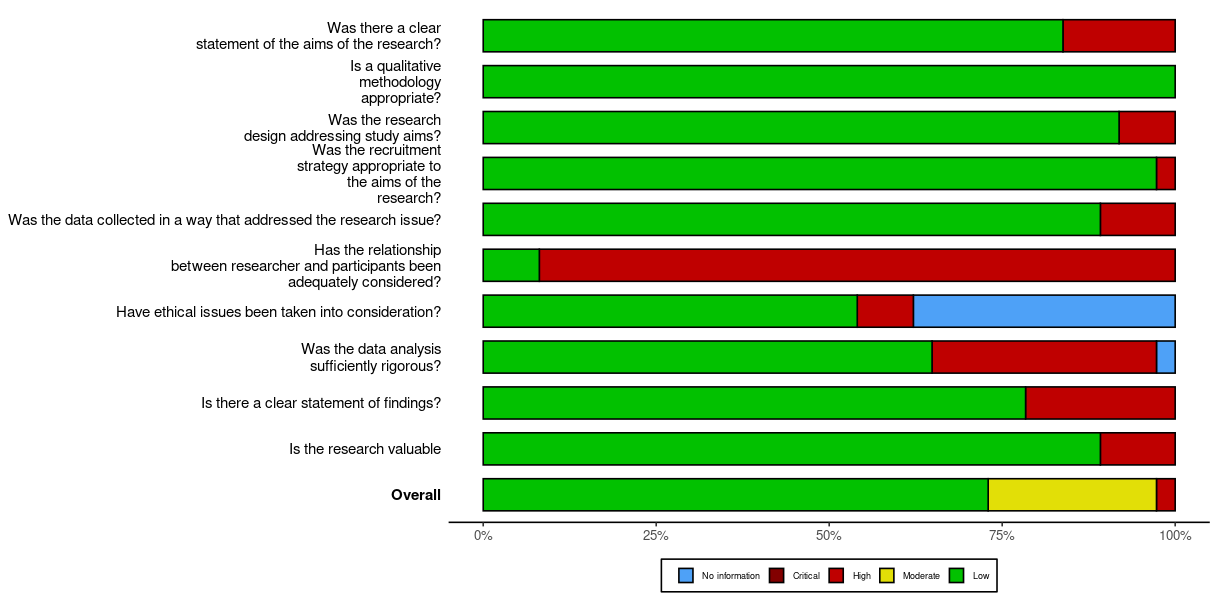
**

# Figure S2: Risk of bias assessment for cross-sectional studies


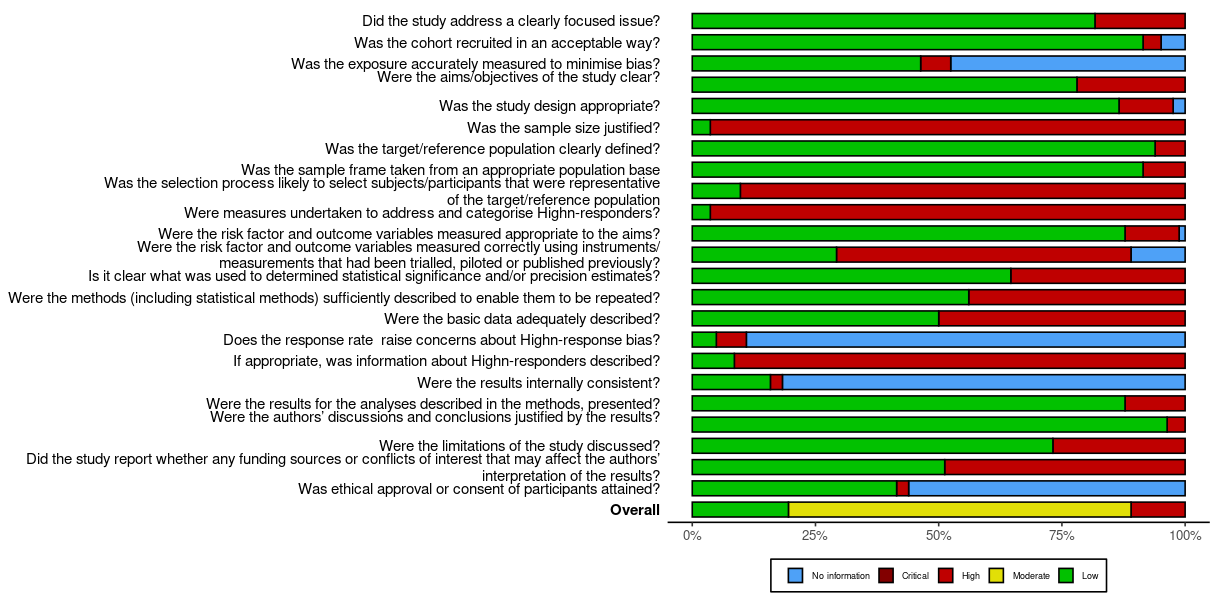


# Figure S3: Risk of bias assessment for cohort studies


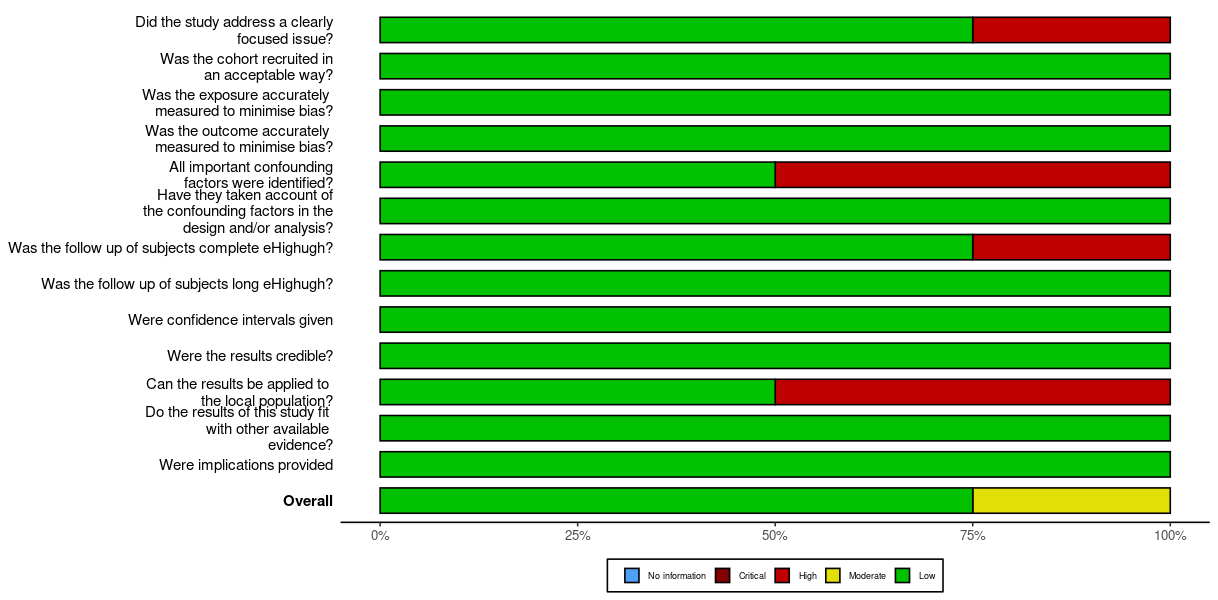


# Figure S4: Risk of bias assessment for mixed-methods studies


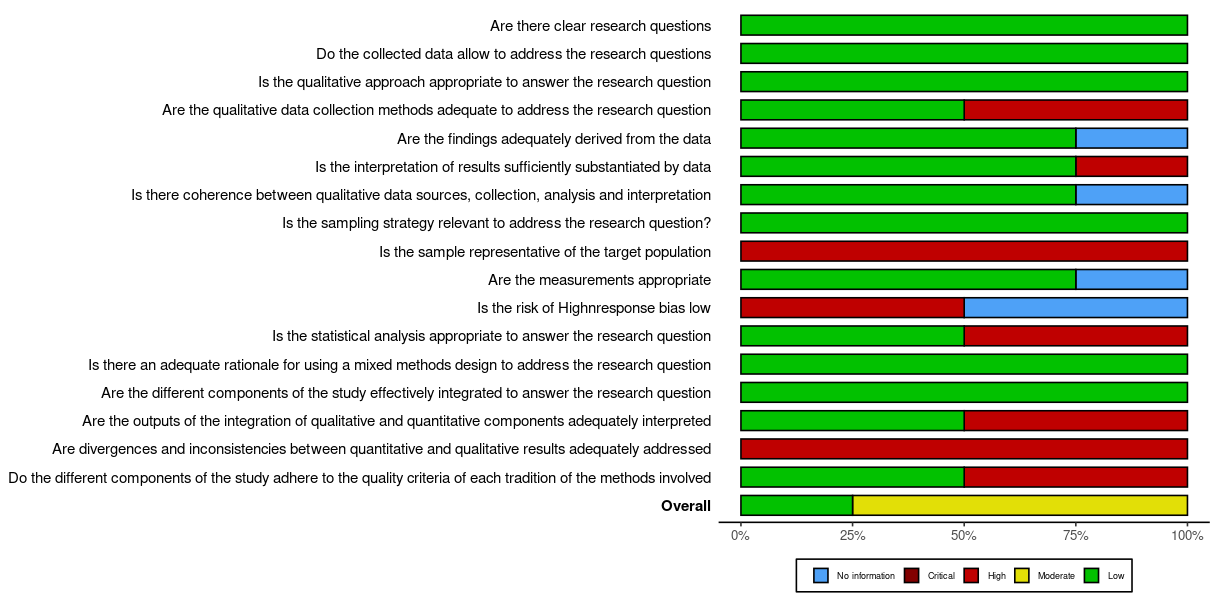


# Table S8: Networks of older adults stratified by population and continent

| Category | Sub-category | Care networks | Number of studies examining care networks |
| --- | --- | --- | --- |
| Minority population | Older adults with disability (n=6) | Family*  Children  Spouse  Other relatives | 5 (83.3%)  3 (50.0%)  2 (33.3%)  2 (33.3%) |
|  |  | Co-residents in a care institute | 2 (33.3%) |
|  |  | Friends | 3 (50.0%) |
|  |  | Neighbours | 2 (33.3%) |
|  |  | Work colleagues | 1 (16.7%) |
|  |  | Church members | 1 (16.7%) |
|  |  | Community | 1 (16.7%) |
|  |  | Health and social care providers | 2 (33.3%) |
|  | Older adults with low socio-economic status (n=2) | Family  Children  Spouse | 1 (50%)  1 (50%)  1 (50%) |
|  |  | Friends | 1 (50%) |
|  |  | Neighbours | 1 (50%) |
|  |  | Community | 2 (100%) |
|  |  | Health and social care providers | 1 (50%) |
|  | LGBTQ older adults (n=3) | Family | 3 (100%) |
|  |  | Friends | 3 (100%) |
|  |  | Relatives | 1 (30.3%) |
|  |  | Community | 1 (30.3%) |
|  |  | Health and social care providers | 1 (30.3%) |
| Continent | North America (n=43) | Family  Children  Spouse  Siblings  Other relatives | 27 (62.8%)  15 (34.9%)  14 (32.6%)  1 (2.3%)  8 (18.6%) |
|  |  | Friends | 22 (51.2%) |
|  |  | Neighbours | 9 (20.9%) |
|  |  | Church members | 4 (9.3%) |
|  |  | Co-residents | 4 (9.3%) |
|  |  | Work colleagues | 2 (4.7%) |
|  |  | Pets | 2 (4.7%) |
|  |  | Community | 15 (34.9%) |
|  |  | Health and social care providers | 11 (25.6%) |
|  | Europe (n=40) | Family  Children  Spouse  Siblings  Other relatives | 32 (80%)  19 (47.5%)  15 (37.5%)  4 (10.0%)  13 (32.5%) |
|  |  | Friends | 25 (62.5%) |
|  |  | Neighbours | 13 (32.5%) |
|  |  | Church members | 3 (7.5%) |
|  |  | Co-residents | 3 (7.5%) |
|  |  | Work colleagues | 2 (5.0%) |
|  |  | Community | 15 (37.5%) |
|  |  | Health and social care providers | 12 (30.0%) |
|  | Asia (n=26) | Family  Children  Spouse  Siblings  Other relatives | 21 (80.8%)  9 (34.6%)  9 (34.6%)  1 (3.8%)  5 (19.2%) |
|  |  | Friends | 17 65,4%) |
|  |  | Neighbours | 5 (19,2%) |
|  |  | Church members | 4 (15.4%) |
|  |  | Work colleagues | 2 (7.7%) |
|  |  | Community | 9 (34.6%) |
|  |  | Health and social care providers | 4 (15.4%) |
|  | Australasia (n=7) | Family  Children  Relatives | 3 (42.8%)  1 (14.3%)  1 (14.3%) |
|  |  | Friends | 5 (71.4%) |
|  |  | Co-residents | 2 (28.6%) |
|  |  | Pets | 1 (14.3%) |
|  |  | Community | 6 (85.7%) |
|  |  | Health and social care providers | 1 (14.3%) |
|  | South America (n=4) | Family  Children  Spouse | 4 (100%)  1 (25%)  2 (50%) |
|  |  | Friends | 3 (75%) |
|  |  | Neighbours | 2 (50%) |
|  |  | Church members | 1 (25%) |
|  |  | Community | 2 (50%) |
|  |  | Health and social care providers | 2 (50%) |
| Time period | 2011-2022 (n=76) | Family  Children  Spouse  Siblings  Other relatives | 60 (78.9%)  34 (44.7%)  33 (43.4%)  4 (5.3%)  20 (26.3%) |
|  |  | Friends | 50 (65.8%) |
|  |  | Neighbours | 18 (23.7%) |
|  |  | Church members | 7 (9,2%) |
|  |  | Work colleagues | 7 (9.2%) |
|  |  | Co-residents | 3 (3.9%) |
|  |  | Community | 30 (39,5%) |
|  |  | Health and social care providers | 16 (21.1%) |
|  | 2001-2010 (n=27) | Family  Children  Spouse  Siblings  Other relatives | 17 (63.0%)  8 (29.6%)  7 (25.9%)  1 (3.7%)  4 (14.8%) |
|  |  | Friends | 14 (51.9%) |
|  |  | Neighbours | 7 (25.9%) |
|  |  | Co-residents | 3 (11.1%) |
|  |  | Church members | 3 (11.1%) |
|  |  | Pets | 1 (3.7%) |
|  |  | Community | 13 (48.1%) |
|  |  | Health and social care providers | 12 (44.4%) |
|  | 1991-2000 (n=11) | Family  Children  Spouse  Siblings  Other relatives | 7 (63.6%)  3 (27.3%)  3 (27.3%)  1 (9.1%)  2 (18.2%) |
|  |  | Friends | 7 (63.6%) |
|  |  | Neighbours | 4 (36.4%) |
|  |  | Church members | 2 (18.2%) |
|  |  | Co-residents | 2 (18.2%) |
|  |  | Pets | 1 (9.1%) |
|  |  | Community | 3 (27.3%) |
|  |  | Health and social care providers | 2 (18.2%) |
|  | 1981-1990 (n=7) | Family  Children  Spouse  Relatives | 4 (57.1%)  1 (14.3%)  1 (14.3%)  1 (14.3%) |
|  |  | Friends | 2 (28.6%) |
|  |  | Neighbour | 2 (28.6%) |
|  |  | Co-resident | 1 (14.3%) |
|  |  | Pets | 1 (14.3%) |
|  |  | Community | 1 (14.3%) |

# References

1. Iacobucci, D., et al., *In Social Network Analysis, Which Centrality Index Should I Use?: Theoretical Differences and Empirical Similarities among Top Centralities.* Journal of Methods and Measurement in the Social Sciences, 2018. **8**(2): p. 72-99.

2. Smith, N.R., et al., *A Guide for Choosing Community Detection Algorithms in Social Network Studies: The Question Alignment Approach.* American journal of preventive medicine, 2020. **59**(4): p. 597-605.

3. Newman, M.E.J. and M. Girvan, *Finding and evaluating community structure in networks.* Physical Review, 2004. **69**(2): p. 1-6.

4. Csardi, G. and T. Nepusz. *Package igraph*. 2022 [cited 2022 June 20]; Available from: <https://cran.r-project.org/web/packages/igraph/igraph.pdf>.

5. Aida, J., et al., *Is social network diversity associated with tooth loss among older Japanese adults?* PloS One, 2016. **11**(7): p. 1-12.

6. Al Kandari, Y.Y., *Relationship of Strength of Social Support and Frequency of Social Contact with Hypertension and General Health Status Among Older Adults in the Mobile Care Unit in Kuwait.* Journal of Cross-Cultural Gerontology, 2011. **26**(2): p. 175-187.

7. Aung, M.N., et al., *A Contemporary Insight into an Age-Friendly Environment Contributing to the Social Network, Active Ageing and Quality of Life of Community Resident Seniors in Japan.* Journal of Aging and Environment, 2021. **35**(2): p. 145-160.

8. Ayalon, L., *Social network type in the continuing care retirement community.* Archives of Gerontology and Geriatrics, 2019. **84**: p. 1-6.

9. Ayalon, L. and V. Green, *Social ties in the context of the continuing care retirement community.* Qualitative Health Research, 2013. **23**(3): p. 396-406.

10. Ayalon, L. and I. Yahav, *Location, location, location: Close ties among older continuing care retirement community residents.* PloS One, 2019. **14**(11): p. 1-17.

11. Baltes, M.M., et al., *On the social ecology of dependence and independence in elderly nursing home residents: a replication and extension.* Journal of Gerontology, 1983. **38**(5): p. 556-564.

12. Barros, E.J.L., S.S.C. Santos, and A.L. Erdmann, *Social network of support for stomized seniors according to complexity.* Acta Paulista de Enfermagem, 2008. **21**(4): p. 595-601.

13. Bear, M., *Social Networks and Health: Impact on Returning Home After Entry into Residential Care Homes.* The Gerontologist, 1990. **30**(1): p. 30-34.

14. Berglund, H., et al., *The Impact of Socioeconomic Conditions, Social Networks, and Health on Frail Older People's Life Satisfaction: A Cross-Sectional Study.* Health Psychology Research, 2016. **4**(1): p. 5578-5578.

15. Bijnsdorp, F.M., et al., *Who provides care in the last year of life?: A description of care networks of community-dwelling older adults in the Netherlands.* BMC Palliative Care, 2019. **18**(1): p. 1-11.

16. Boneham, M.A. and J.A. Sixsmith, *The voices of older women in a disadvantaged community: Issues of health and social capital.* Social Science & Medicine 2006. **62**(2): p. 269-279.

17. Brennan-Ing, M., et al., *Social Care Networks and Older LGBT Adults: Challenges for the Future.* Journal of Homosexuality, 2014. **61**(1): p. 21-52.

18. Brito, T.R.P.d., et al., *Social network and older people's functionality: Health, Well-being, and Aging (SABE) study evidences.* Revista Brasileira de Epidemiologia, 2019. **21**(2): p. 1-15.

19. Buckley, C. and G. McCarthy, *An Exploration of Social Connectedness as Perceived by Older Adults in a Long-Term Care Setting in Ireland.* Geriatric Nursing 2009. **30**(6): p. 390-396.

20. Canham, S.L., et al., *Contextual Factors for Aging Well: Creating Socially Engaging Spaces Through the Use of Deliberative Dialogues.* The Gerontologist, 2018. **58**(1): p. 140-148.

21. Carpenter, B.D., *Family, Peer, and Staff Social Support in Nursing Home Patients: Contributions to Psychological Well-Being.* Journal of applied gerontology, 2002. **21**(3): p. 275-293.

22. Coe, R.M., et al., *Elderly persons without family support networks and use of health services: a follow-up report on social network relationships.* Research on Aging, 1985. **7**(4): p. 617-622.

23. Coe, R.M., et al., *Complementary and Compensatory Functions in Social Network Relationships Among the Elderly.* The Gerontologist, 1984. **24**(4): p. 396-400.

24. Cohen, A.L. and C.R. Bennett, *Support Network Connectedness in the Lives of Community-Dwelling Rural Elders and Their Families.* Marriage & Family Review, 2017. **53**(6): p. 576-588.

25. Cott, C.A., et al., *Helping Networks in Community Home Care for the Elderly: Types of Team.* Canadian Journal of Nursing Research, 2008. **40**(1): p. 18-37.

26. Crooks, V.C., et al., *Social Network, Cognitive Function, and Dementia Incidence Among Elderly Women.* American Journal of Public Health, 2008. **98**(7): p. 1221-1227.

27. Dagnan, D. and L. Ruddick, *The Social Networks of Older People with Learning Disabilities Living in Staffed Community Based Homes.* The British journal of developmental disabilities, 1997. **43**(84): p. 43-53.

28. Doekhie, K., et al., *Elderly patients’ decision-making embedded in the social context: a mixed-method analysis of subjective norms and social support.* BMC Geriatrics, 2020. **20**(1): p. 53-53.

29. Doubova, S.V., P. Espinosa-Alarcán, and S. Flores-Hernndez, *Social network types and functional dependency in older adults in Mexico.* BMC Public Health, 2010. **10**(1): p. 104-104.

30. Drennan, J., et al., *Support networks of older people living in the community.* International Journal of Older People Nursing, 2008. **3**(4): p. 234-242.

31. Duner, A. and M. Nordstrom, *The roles and functions of the informal support networks of older people who receive formal support: a Swedish qualitative study.* Ageing and Society, 2007. **27**(1): p. 67-85.

32. Dupuis-Blanchard, S., A. Neufeld, and V.R. Strang, *The Significance of Social Engagement in Relocated Older Adults.* Qualitative Health Research, 2009. **19**(9): p. 1186-1195.

33. Ehrlich, P., *Informal Support Networks Meet Health needs of Rural Elderly.* Journal of Gerontological Social Work, 1985. **9**(1): p. 85-98.

34. Evans, N., et al., *Social support and care arrangements of older people living alone in rural Malaysia.* Ageing and Society, 2018. **38**(10): p. 2061-2081.

35. Fernandez‐Carro, C. and A. Vlachantoni, *The role of social networks in using home care by older people across Continental Europe.* Health & Social Care in the Community, 2019. **27**(4): p. 936–952.

36. Gallo, F., *Social support networks and the health of elderly persons.* National Association of Social Workers, 1984. **20**(4): p. 13-19.

37. Giles, L.C., et al., *Do social networks affect the use of residential aged care among older Australians?* BMC Geriatrics, 2007. **7**(24): p. 1-10.

38. Golden, J., R.M. Conroy, and B.A. Lawlor, *Social support network structure in older people: Underlying dimensions and association with psychological and physical health.* Psychology, health & medicine, 2009. **14**(3): p. 280-290.

39. Golden, J., et al., *Loneliness, social support networks, mood and wellbeing in community-dwelling elderly.* International Journal of Geriatric Psychiatry, 2009. **24**(7): p. 694-700.

40. Greaves, C.J. and L. Farbus, *Effects of creative and social activity on the health and well-being of socially isolated older people: outcomes from a multi-method observational study.* The journal of the Royal Society for the Promotion of Health, 2006. **126**(3): p. 134-142.

41. Green, M., *Do the companionship and community networks of older LGBT adults compensate for weaker kinship networks?* Quality in Ageing and Older Adults, 2016. **17**(1): p. 36-49.

42. Gu, L., M.W. Rosenberg, and J. Zeng, *Changing caregiving relationships for older home-based Chinese people in a transitional stage: Trends, factors and policy implications.* Archives of Gerontology and Geriatrics, 2017. **70**: p. 219-229.

43. Jacobs, M.T., et al., *Diversity in older adults’ care networks: the added value of individual beliefs and social network proximity.* The Journal of Gerontology, 2018. **73**(2): p. 326-336.

44. Jacobs, M., et al., *Linkages between informal and formal care-givers in home-care networks of frail older adults.* Ageing and Society, 2016. **36**(8): p. 1604-1624.

45. Kuiper, J.S., et al., *A longitudinal study of the impact of social network size and loneliness on cognitive performance in depressed older adults.* Aging & Mental Health, 2020. **24**(6): p. 889-897.

46. Lee, W.K.-m., *Living Arrangements and Informal Support for the Elderly: Alteration to Intergenerational Relationships in Hong Kong.* Journal of Intergenerational Relationships, 2004. **2**(2): p. 27-49.

47. Loeb, S.J., et al., *Supporting Older Adults Living With Multiple Chronic Conditions.* Western Journal of Nursing Research, 2003. **25**(1): p. 8-29.

48. Li, H., D. Edwards, and N. Morrow-Howell, *Informal Caregiving Networks and Use of Formal Services by Inner-City African American Elderly with Dementia.* Families in Society, 2004. **85**(1): p. 55-62.

49. Li, M., X. Dong, and D. Kong, *Social Networks and Depressive Symptoms among Chinese Older Immigrants: Does Quantity, Quality, and Composition of Social Networks Matter?* Clinical Gerontologist, 2021. **44**(2): p. 181-191.

50. Litwin, H., *The Provision of Informal Support by Elderly People Residing in Assisted Living Facilities.* The Gerontologist, 1998. **38**(2): p. 239-246.

51. Litwin, H., *Support Network Type and Patterns of Help Giving and Receiving Among Older People.* Journal of Social Service Research, 1999. **24**(3-4): p. 83-101.

52. Liu, X., G. Cook, and M. Cattan, *Support networks for Chinese older immigrants accessing English health and social care services: the concept of Bridge People.* Health & Social Care in the Community, 2017. **25**(2): p. 667-677.

53. McDonald, R.M. and P.J. Brown, *Exploration of social support systems for older adults: A preliminary study.* Contemporary Nurse, 2008. **29**(2): p. 184-194.

54. McFarland, M.L., *The effect of the provision of in home services on the elderly person's informal support network*, in *Social Work*. 1991, University of Maryland: Baltimore. p. 1-218.

55. McLeod, E., et al., *For the Sake of their Health: Older Service Users’ Requirements for Social Care to Facilitate Access to Social Networks Following Hospital Discharge.* The British Journal of Social Work, 2008. **38**(1): p. 73-90.

56. Neves, B.B., et al., *Can Digital Technology Enhance Social Connectedness Among Older Adults? A Feasibility Study.* Journal of Applied Gerontology, 2019. **38**(1): p. 49-72.

57. Rodríguez, M., M.Á. Minguela Recover, and J.A. Camacho Ballesta, *The importance of the size of the social network and residential proximity in the reception of informal care in the European Union.* European Journal of Social Work, 2018. **21**(5): p. 653-664.

58. Moorman, S.M. and K. Boerner, *How Social Network Size and Quality Affect End-of-Life Surrogate Preferences.* Journal of Gerontology, 2018. **73**(4): p. 704-712.

59. Nielson, L., J. Wiles, and A. Anderson, *Social exclusion and community in an urban retirement village.* Journal of Aging Studies, 2019. **49**: p. 25-30.

60. Oh, A., et al., *Social Support and Patterns of Institutionalization Among Older Adults: A Longitudinal Study.* Journal of the American Geriatrics Society, 2019. **67**(12): p. 2622-2627.

61. Vos-den Ouden, W., et al., *The impact of social network change and health decline: A qualitative study on experiences of older adults who are ageing in place.* BMC Geriatrics, 2021. **21**(480): p. 1-13.

62. Park, S., J.Y. Kang, and L.A. Chadiha, *Social Network Types, Health, and Health-Care Use Among South Korean Older Adults.* Research on Aging, 2018. **40**(2): p. 131-154.

63. Peek, C.W., B.A. Zsembik, and R.T. Coward, *The Changing Caregiving Networks of Older Adults.* Research on Aging, 1997. **19**(3): p. 333-361.

64. Pleschberger, S. and P. Wosko, *From neighbour to carer: An exploratory study on the role of non-kin-carers in end-of-life care at home for older people living alone.* Palliative Medicine, 2017. **31**(6): p. 559-565.

65. Powers, B.A.J.A.A.i.N.S., *Social networks, social support, and elderly institutionalized people.* Advances in Nursing Science, 1988. **10**(2): p. 40-58.

66. Powers, B.A., *Relationships Among Older Women Living in a Nursing Home.* Journal of Women & Aging, 1996. **8**(3-4): p. 179-198.

67. Powers, B.A., *The roles staff play in the social networks of elderly institutionalized people.* Social Science & Medicine, 1992. **34**(12): p. 1335-1343.

68. Prosser, L., M. Townsend, and P. Staiger, *Older people's relationships with companion animals: a pilot study.* Nursing Older People, 2008. **20**(3): p. 29-32.

69. Reed, C.J., *Social Exchanges of Older Women in Assisted Living Settings*, in *School of Social Work*. 2006, University of Kansas ProQuest. p. 1-139.

70. Rennemark, M. and B. Hagberg, *Gender specific associations between social network and health behavior in old age.* Aging & Mental Health, 1999. **3**(4): p. 320-327.

71. Roberts, T.J., *Nursing home resident relationship types: What supports close relationships with peers & staff?* Journal of Clinical Nursing, 2018. **27**(23-24): p. 4361-4372.

72. Rocha, S.M.M., M.L. Nogueira, and M. Cesario, *Social support and networks in health promotion of older people: a case study in Brazil.* International Journal of Older People Nursing, 2009. **4**(4): p. 288-298.

73. Roe, B., et al., *Elders' needs and experiences of receiving formal and informal care for their activities of daily living.* Journal of Clinical Nursing, 2001. **10**(3): p. 389-397.

74. Rowe, J.L., et al., *Social Support and Suicidal Ideation in Older Adults Using Home Healthcare Services.* The American Journal of Geriatric Psychiatry, 2006. **14**(9): p. 758-766.

75. Saito, T., et al., *Influence of social relationship domains and their combinations on incident dementia: a prospective cohort study.* Journal of Epidemiology and Community Health, 2018. **72**(1): p. 7-12.

76. Schenk, N., et al., *Older adults’ networks and public care receipt: do partners and adult children substitute for unskilled public care?* Ageing and Society, 2014. **34**(10): p. 1711-1729.

77. Schmidt, T., et al., *Social network characteristics as correlates and moderators of older adults’ quality of life—the SHARE study.* European Journal of Public Health, 2021. **31**(3): p. 541-547.

78. Schnettler, S. and T. WÖHler, *No children in later life, but more and better friends? Substitution mechanisms in the personal and support networks of parents and the childless in Germany.* Ageing and Society, 2016. **36**(7): p. 1339-1363.

79. Siette, J., et al., *Social networks and cognitive function in older adults receiving home- and community-based aged care.* Archives of Gerontology and Geriatrics, 2020. **89**: p. 1-7.

80. Sintonen, S. and A. Pehkonen, *Effect of social networks and well-being on acute care needs.* Health & Social Care in the Community, 2014. **22**(1): p. 87-95.

81. Spillman, B.C., et al., *Change Over Time in Caregiving Networks for Older Adults With and Without Dementia (vol 75, pg 1563, 2020).* Journals of Gerontology, 2020. **75**(7): p. 1563-1572.

82. Stacey-Konnert, C. and J. Pynoos, *Friendship and Social Networks in a Continuing Care Retirement Community.* Journal of Applied Gerontology, 1992. **11**(3): p. 298-313.

83. Stafford, M., et al., *Social connectedness and engagement in preventive health services: an analysis of data from a prospective cohort study.* The Lancet. Public health, 2018. **3**(9): p. e438-e446.

84. Sta Maria, M.A., A.A.C. Bonanza, and P.A.S. Arceg, *Quality of support in the social networks of older Filipino church members: an exploratory study.* Quality in Ageing, 2018. **19**(1): p. 42-53.

85. Palo Stoller, E. and K.L. Pugliesi, *Size and Effectiveness of Informal Helping Networks: A Panel Study of Older People in the Community.* Journal of Health and Social Behavior, 1991. **32**(2): p. 180-191.

86. Suanet, B., M.I. Broese Van Groenou, and T.G. Van Tilburg, *Social network type and informal care use in later life: a comparison of three Dutch birth cohorts aged 75–84.* Ageing and Society, 2019. **39**(4): p. 749-770.

87. Sullivan, J.L., et al., *Social Connection and Psychosocial Adjustment among Older Male Veterans Who Return to the Community from VA Nursing Homes.* Clinical Gerontologist, 2021. **44**(4): p. 450-459.

88. Tang, F. and Y. Lee, *Social Support Networks and Expectations for Aging in Place and Moving.* Research on Aging, 2011. **33**(4): p. 444-464.

89. Teerawichitchainan, B., W. Pothisiri, and G.T. Long, *How do living arrangements and intergenerational support matter for psychological health of elderly parents? Evidence from Myanmar, Vietnam, and Thailand.* Social Science & Medicine 2015. **136-137**: p. 106-116.

90. Tolkacheva, N., et al., *The impact of informal care-giving networks on adult children's care-giver burden.* Ageing and Society, 2011. **31**(1): p. 34-51.

91. Verbeke, A., *Lonely last days? Social networks and formal care at the deathbed of urban elderly in Antwerp, Brussels and Ghent, 1797.* The History of the Family, 2021. **26**(1): p. 123-148.

92. Vos, W.H., et al., *Exploring the impact of social network change: Experiences of older adults ageing in place.* Health & Social Care in the Community, 2019. **28**(1): p. 116-126.

93. Wenger, G.C., *The formation of social networks: Self help, mutual aid, and old people in contemporary Britain.* Journal of Aging Studies, 1993. **7**(1): p. 25-40.

94. Wiersma, E.C. and A. Pedlar, *The Nature of Relationships in Alternative Dementia Care Environments.* Canadian Journal on Aging, 2008. **27**(1): p. 101-108.

95. Wiles, J., et al., *Befriending Services for Culturally Diverse Older People.* Journal of Gerontological Social Work, 2019. **62**(7): p. 776-793.

96. Williams, S.W. and P. Dilworth-Anderson, *Systems of Social Support in Families Who Care for Dependent African American Elders.* The Gerontologist, 2002. **42**(2): p. 224-236.

97. Wu, F. and Y. Sheng, *Social support network, social support, self-efficacy, health-promoting behavior and healthy aging among older adults: A pathway analysis.* Archives of Gerontology and Geriatrics, 2019. **85**: p. 1-6.

98. Xu, L., et al., *Family Relationships, Friend Network, and Worry: A Comparison Among Chinese Older Adults in Immigrant, Transnational, and Nonmigrant Families.* Journal of Ethnic & Cultural Diversity in Social Work, 2019. **28**(3): p. 317-333.

99. Yoo, J.A. and A. Zippay, *Social networks among lower income Korean elderly immigrants in the U.S.* Journal of Aging Studies, 2012. **26**(3): p. 368-376.

100. Zhang, D., et al., *The moderating effect of social support on the relationship between physical health and suicidal thoughts among Chinese rural elderly: A nursing home sample.* International Journal of Mental Health Nursing, 2018. **27**(5): p. 1371-1382.

101. Cheng, G.H.L., et al., *Transitions between social network profiles and their relation with all-cause mortality among older adults.* Social Science & Medicine, 2022. **292**(1): p. 1-8.

102. Cohen, C.I., *Social networks and residential status in community-dwelling older adults with schizophrenia: Compensation by reconfiguration?* The American Journal of Geriatric Psychiatry, 2022. **30**(11): p. 1159-1167.

103. Cohn-Schwartz, E., M. Levinsky, and H. Litwin, *Social network type and subsequent cognitive health among older Europeans.* International Psychogeriatrics, 2021. **33**(5): p. 495-504.

104. York Cornwell, E. and A.W. Goldman, *Local Ties in the Social Networks of Older Adults.* The Journals of Gerontology, 2021. **76**(4): p. 790-800.

105. Domènech-Abella, J., et al., *Social network size, loneliness, physical functioning and depressive symptoms among older adults: Examining reciprocal associations in four waves of the Longitudinal Aging Study Amsterdam (LASA).* International journal of geriatric psychiatry, 2021. **36**(10): p. 1541-1549.

106. Guadalupe, S. and H.T. Vicente, *Types of personal social networks of older adults in Portugal.* Social Indicators Research, 2022. **160**(2-3): p. 445-466.

107. Hamlin, A.M., et al., *Social Engagement and Its Links to Cognition Differ Across Non-Hispanic Black and White Older Adults.* Neuropsychology, 2022. **36**(7): p. 640-650.

108. Holcomb, J.L., et al., *A qualitative study examining the social network types of older sexual and gender minority (SGM) women and gender non-binary adults.* Journal of Gay & Lesbian Social Services, 2022. **34**(1): p. 1-20.

109. Kim, H., et al., *Social network characteristics predict loneliness in older adults.* Gerontology, 2022. **68**(3): p. 309-320.

110. Litwin, H. and M. Levinsky, *Social networks and mental health change in older adults after the Covid-19 outbreak.* Aging & Mental Health, 2022. **26**(5): p. 925-931.

111. Lottmann, R. and A. King, *Who can I turn to? Social networks and the housing, care and support preferences of older lesbian and gay people in the UK.* Sexualities, 2022. **25**(1-2): p. 9-24.

112. McCausland, D., et al., *The nature and quality of friendship for older adults with an intellectual disability in Ireland.* Journal of Applied Research in Intellectual Disabilities, 2021. **34**(3): p. 763-776.

113. Meister, L.M. and L.B. Zahodne, *Associations between social network components and cognitive domains in older adults.* Psychology and Aging, 2022. **37**(5): p. 591-603.

114. Nie, Y., et al., *Social networks and cognitive function in older adults: findings from the HAPIEE study.* BMC Geriatrics, 2021. **21**(1): p. 1-14.

115. Park, N.S., et al., *The role of social networks on depressive symptoms: A comparison of older koreans in three geographic areas.* International Journal of Aging & Human Development, 2021. **92**(3): p. 364-382.

116. Parkhurst, K.A., et al., *Social network subtypes among socially disconnected older adults at risk for suicide: A latent class analysis.* Suicide & life-threatening behavior, 2022. **52**(5): p. 963-974.

117. Rhee, T.G., R.A. Marottoli, and J.K. Monin, *Diversity of social networks versus quality of social support: Which is more protective for health-related quality of life among older adults?* Preventive Medicine, 2021. **145**(1): p. 1-19.

118. Saito, T., et al., *Association between intra-individual changes in social network diversity and global cognition in older adults: Does closeness to network members make a difference?* Journal of Psychosomatic Research, 2021. **151**(1): p. 110658-110658.

119. Stephens, C. and H. Phillips, *Older People’s Neighborhood Perceptions Are Related to Social and Emotional Loneliness and Mediated by Social Network Type.* The Gerontologist, 2022. **62**(9): p. 1336-1346.

120. Stipkova, M., *Marital status, close social network and loneliness of older adults in the Czech Republic.* Ageing and Society, 2021. **41**(3): p. 671-685.

121. Sung, P., et al., *Transitions in social network types over time among older adults.* Gerontology, 2022. **68**(7): p. 1-12.

122. Torres, Z., A. Oliver, and J.M. Tomás, *Mapping protective performance of social network types on health and quality of life in older people in European regions.* Journal of Aging and Health, 2022. **1**(1): p. 89826432211420-8982643221142078.

123. Xin, Y. and D. Li, *Impacts of psychological resources, social network support and community support on social participation of older adults in China: Variations by different health‐risk groups.* Health & Social Care in the Community, 2022. **30**(5): p. e2340-e2349.

124. Yoo-Jeong, M., A.L. Nguyen, and D. Waldrop, *Social network size and its relationship to domains of quality-of-life among older persons living with HIV.* AIDS Care, 2022. **1**(1): p. 1-8.

125. Zhang, Q. and Z. Li, *The impact of internet use on the social networks of the elderly in China-the mediating effect of social participation.* International Journal of Environmental Research and Public Health, 2022. **19**(15): p. 1-17.

126. York Cornwell, E., A.W. Goldman, and M. Schafer, *Local ties in the social networks of older adults.* The Journals of Gerontology, 2021. **76**(4): p. 790-800.
